# CREATE and CONNECT: Arboviruses at the intersection of research and community outreach

**DOI:** 10.1371/journal.pntd.0013436

**Published:** 2025-08-22

**Authors:** Daniel Jacob, Marcelle Alves de Oliveira, Mikaelly Frasson Biccas, Ana Maria de Oliveira Paschoal, Anna Catarina Dias Soares Guimarães, Samantha Stephany Fiuza Meneses Viegas, Thaís Alkifeles Costa, Gabriela Fernanda Garcia-Oliveira, Gabriel Dias Moreira, Matheus Soares Arruda, Nadja Simbera Hemetrio, Marina do Vale Beirão, Kathryn A. Hanley, Nikos Vasilakis, Betânia Paiva Drumond

**Affiliations:** 1 Department of Microbiology, Universidade Federal de Minas Gerais, Belo Horizonte, Minas Gerais, Brazil; 2 Fundação de Parques Municipais e Zoobotânica, Prefeitura de Belo Horizonte, Belo Horizonte, Minas Gerais, Brazil; 3 Department of Biology, New Mexico State University, Las Cruces, New Mexico, United States of America; 4 Department of Pathology, University of Texas Medical Branch at Galveston, Galveston, Texas, United States of America; 5 Center for Vector-Borne and Zoonotic Diseases, University of Texas Medical Branch at Galveston, Galveston, Texas, United States of America; 6 Institute for Human Infection and Immunity, University of Texas Medical Branch at Galveston, Galveston, Texas, United States of America; NIAID Integrated Research Facility, UNITED STATES OF AMERICA

Neglected tropical diseases (NTDs) are a diverse group of illnesses caused by viruses, bacteria, parasites, fungi, and toxins resulting in significant health, social, and economic burdens. NTDs predominantly impact impoverished communities in tropical regions, and some NTDs extend beyond these areas, presenting a wider global health concern. Among these diseases, the ones caused by arthropod-borne viruses (arboviruses) represent a serious global public health problem [1]. In Brazil, the four dengue serotypes (DENV-1 to DENV-4), Zika (ZIKV), and chikungunya (CHIKV) viruses are primarily transmitted by anthropophilic mosquitoes such as *Aedes aegypti*, causing significant epidemiological and disease burden [2]. Additionally, the yellow fever virus (YFV) remains a critical concern. After the urban cycle of yellow fever (YF) was eradicated in 1942, it persisted in an endemic, sylvatic cycle between non-human primates (NHPs), and vectors such as *Sabethes* and *Haemagogus* mosquitoes in the Amazon basin, where it spilled over occasionally into humans. This period of relative quiet ended in 2016 with a massive outbreak of YFV in eastern Brazil in both NHPs and humans. The state of Minas Gerais faced the most significant YF outbreaks in the last 70 years, with deaths of NHPs in the metropolitan region of Belo Horizonte (BH) [3,4] (Fig A in S1 File). YFV has been detected in Minas Gerais state, with recent infections in humans and NHPs [5–8].

As part of the NIH-funded initiative, following YFV activity, the Coordinating Research on Emerging Arboviral Threats Encompassing the Neotropics (CREATE-NEO) [9], has been conducting surveillance of NHPs and mosquitoes in urban parks in BH to monitor YFV activity. In parallel, we developed an interactive exhibition and educational materials to be presented in the urban parks across BH, with the aim of promoting scientific outreach and enhancing public understanding of arboviruses, including their transmission, and prevention. The exhibition “CREATE and CONNECT” was designed for diverse audiences, ranging from children to adults. The exhibition was divided into three thematic units: (i) arboviruses: understanding viral structures and transmission cycles; (ii) vectors: observing mosquito morphology and their role in virus transmission; and (iii) hosts: exploring NHPs as sentinel species for YFV and promoting responsible wildlife interactions. The material for the exhibition was prepared by CREATE-NEO researchers and students using affordable materials.

## The exhibition CREATE and CONNECT

**Fig 1:**
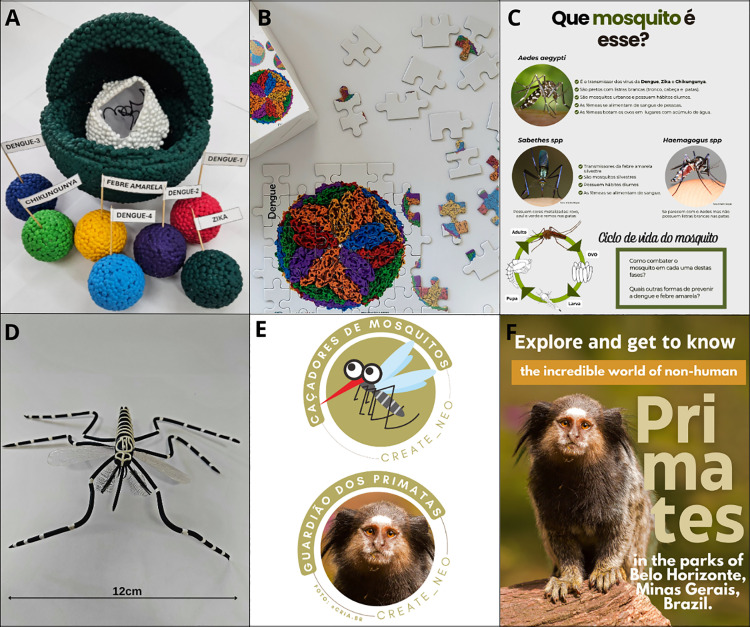
CREATE and CONNECT. **A**: Virus models using Styrofoam and water-based paint were constructed and illustrated the arbovirus particle (bigger model) with envelope in green, capsid in white, and RNA in black. Colored smaller models illustrate DENV-1 to 4, ZIKV, CHIKV, and YFV. **B**: Puzzle showing the ultrastructure of DENV-3 [10]. **C**: The poster “Which mosquito is this?” illustrate domestic and sylvatic mosquitoes, their characteristics and habits [11,12]. **D**: An air-dry clay model of *Ae. aegypti*. **E**: Reward stickers for children (Mosquito Hunters [13] and Primate guardian). **F**: Cover of the e-book “Primates in parks of BH”. Parts of the material (specifically C and E,) were created using freely available images sourced from the internet under open-access terms (https://commons.wikimedia.org/wiki/File:CDC-Gathany-Aedes-albopictus-1.jpg [11], https://openclipart.org/detail/324257/life-cycle-of-the-mosquito [12], https://openclipart.org/detail/315188/mosquito-5 [13]).

The exhibition began in the arbovirus sector, where arboviruses, their structures, and transmission cycles were presented. Special attention was given to discussing the relevance of vaccination against dengue and YF. Styrofoam spheres of different sizes and water-based paint were used to create colored viral models, illustrating the simplicity and diversity of arboviruses (Fig 1A). A puzzle showing the ultramicroscopic structure of DENV-3 also illustrated the virus structure [10] (Fig 1B). Several visitors reported not knowing the existence of four serotypes and that they could be infected more than once with DENV. To illustrate how arboviruses are transmitted, whether in urban or sylvatic cycles, a banner was prepared and displayed (Fig B in S1 File), showing how mosquitoes become infected and subsequently transmit the virus to other hosts. Following this explanation, prevention strategies were discussed, including the importance of vaccination against DENV and YFV, as well as the use of personal protective measures to avoid mosquito bites. It was common for parents or children to ask several questions about vaccines and how they work. They also inquired about the symptoms and how to treat DENV, YFV, ZIKV, and CHIKV.

In the entomological unit, visitors could observe pictures of mosquitoes, such as *A. aegypti*, *Sabethes* spp., and *Haemagogus* spp., highlighting the morphological and ecological differences between urban and sylvatic mosquitoes (Fig 1C). To better understand the morphology of mosquitoes, a model of *A. aegypti* (made with air-dry clay) (Fig 1D), and a diagram showing mosquito morphology (Fig C in S1 File) were used to show the parts of the mosquito and how mosquitoes become infected and transmit arboviruses when biting a person. As a by-product of our fieldwork, mosquito specimens were preserved (Fig D in S1 File) and used for observation using hand-held magnifying glasses or a stereomicroscope. This activity aroused great interest and enthusiasm among the visitors, who reported that they could not have imagined how different mosquitoes are, their detailed morphology, and their potential beauty. A diagram showing the life cycle of mosquitoes was used to emphasize how the community can contribute to mosquito control (Fig 1C). In a *spot-the-difference* game, visitors put into practice their knowledge to avoid standing water in a domestic environment to combat the proliferation of *A. aegypti*. Simulating the work of entomologists during fieldwork, visitors learned how to capture mosquitoes with traps and nets (Fig E in S1 File). Lastly, children played with mosquito puzzles (Fig F in S1 File) and received the “mosquito hunter” sticker (Fig 1E).

Finally, the third unit focused on wild hosts of YFV, highlighting their crucial role as sentinels for YF and demystifying their ability to transmit the virus to humans. Black-tufted marmosets (*Callithrix penicillata*) and the capuchin monkey (*Sapajus nigritus*) are found in parks in BH. Characteristics and habits of marmosets (Fig G in S1 File) and capuchin monkeys were presented in posters, folders (S2 File) and in an educational book (Fig 1F, S2 File). We raised awareness among visitors about responsible interactions with wildlife, warning of the risks of feeding wild animals, including the transmission of viruses such as rabies virus and herpesviruses. Memory games (Fig H in S1 File) and models illustrated proper diet (natural fruits, eggs, insects, tree gum), and inadequate food for NHPs (commercial fruits, biscuits, popcorn). Most visitors reported the habit of feeding wild animals in parks with fruits, biscuits, etc. and noted that, given what they had learned with the exhibition, they would no longer feed wild animals in the parks. The visitors were invited to observe the NHPs from a distance using binoculars, children played with primate puzzles (Fig H in S1 File) and received the “primate guardian” sticker (Fig 1E).

Overall, 15 exhibitions were held in 2023 and 2024, reaching approximately 985 participants. The diverse audience included children, teenagers, adults, and educators. The exhibition was hosted in collaboration with the Municipal Parks and Zoobotanical Foundation of Belo Horizonte as part of the “Ambientar Project,” which promotes environmental education in city parks. Exhibitions were held in eight parks/green areas in BH (Fig A in S1 File), where arbovirus surveillance has been carried out during the CREATE-NEO project [9]. The exhibition also took place during scientific conferences and community outreach events in four cities of Minas Gerais (Monte Azul, Grão Mogol, Botumirim, and Montes Claros) (Fig A in S1 File).

## Insights and perspectives

The exhibition aroused great interest and engagement, with visitors praising the learning experience spontaneously. The adults participated actively while the children engaged in the games and playful activities. The positive feedback from visitors reinforced the success of the exhibition. The researchers had an essential role in adapting the language for different audiences, explaining the units of the exhibition, answering questions, and reinforcing preventive measures against YFV and other arboviruses.

Community science outreach events are essential for promoting public understanding of scientific concepts and adherence to public health measures. These events bridge the gap between researchers and the wider community, making complex scientific concepts accessible for people of all ages and backgrounds. The “CREATE and CONNECT” exhibition showed that simple and accessible hands-on activities, interactive demonstrations, and open discussions can promote scientific awareness of population.

Moreover, dissemination events can inspire the next generation of scientists and encourage critical thinking. This type of activity is highly useful and easily adaptable for K-12 teachers and students, providing an engaging, affordable hands-on approach to science education. Teachers can incorporate similar activities in the classroom to illustrate viral structures, disease transmission, and prevention methods, making abstract concepts more real and easier to understand. Educators can foster scientific curiosity and public health awareness from an early age using these activities into biology, environmental science, and health education classes, empowering students to apply their knowledge to real-world issues. Moreover, students can actively participate by creating materials and presenting exhibitions during classes, science fairs, and community events.

The CREATE and CONNECT outreach model can be effectively adapted to raise awareness and improve community engagement for other NTDs. By tailoring educational materials to specific diseases and regional contexts, CREATE and CONNECT could serve as a scalable framework for NTD awareness campaigns, fostering collaboration between researchers, educators, and local populations and allowing communities to take proactive measures in the prevention and control of diseases. Activities like this are essential for integrating social, health, and environmental efforts to expand the prevention and control of neglected tropical diseases.

## Supporting information

S1 File **Fig A.** Cities Where “CREATE and CONNECT” Exhibition Took Place in 2023 and 2024, Brazil. **Fig B.** Arbovirus transmission. **Fig C.** Mosquitoes anatomy sketch. **Fig D.** Entomological collection of mosquito vectors of arboviruses. **Fig E.** Trap and hand nets for mosquito sampling. **Fig F.** Mosquito puzzle. **Fig G.** Black-tufted marmoset poster. **Fig H.** Memory game **(A)** and Marmoset puzzle **(B)**.
(S1_File.PDF)

S2 File Folder distributed to visitors and Educational Book. (S2_File.PDF)
