# Supplementary material for: CREATE and CONNECT: Arboviruses at the intersection of research and community outreach
Source: PLoS Negl Trop Dis. 2025 Aug 22;19(8):e0013436. doi: 10.1371/journal.pntd.0013436 (PMC12483539; doi:10.1371/journal.pntd.0013436)
Supplement: S1 File — Fig A. Cities Where “CREATE and CONNECT” Exhibition Took Place in 2023 and 2024, Brazil. Fig B. Arbovirus transmission. Fig C. Mosquitoes anatomy sketch. Fig D. Entomological collection of mosquito vectors of arboviruses. Fig E. Trap and hand nets for mosquito sampling. Fig F. Mosquito puzzle. Fig G. Black-tufted marmoset poster. Fig H. Memory game (A) and Marmoset puzzle (B). (S1_File.PDF) [file pntd.0013436.s001.pdf]

S1 File – Supplementary figures

CREATE and CONNECT - Arboviruses at the intersection of Research and Community Outreach

Jacob et al 2025.

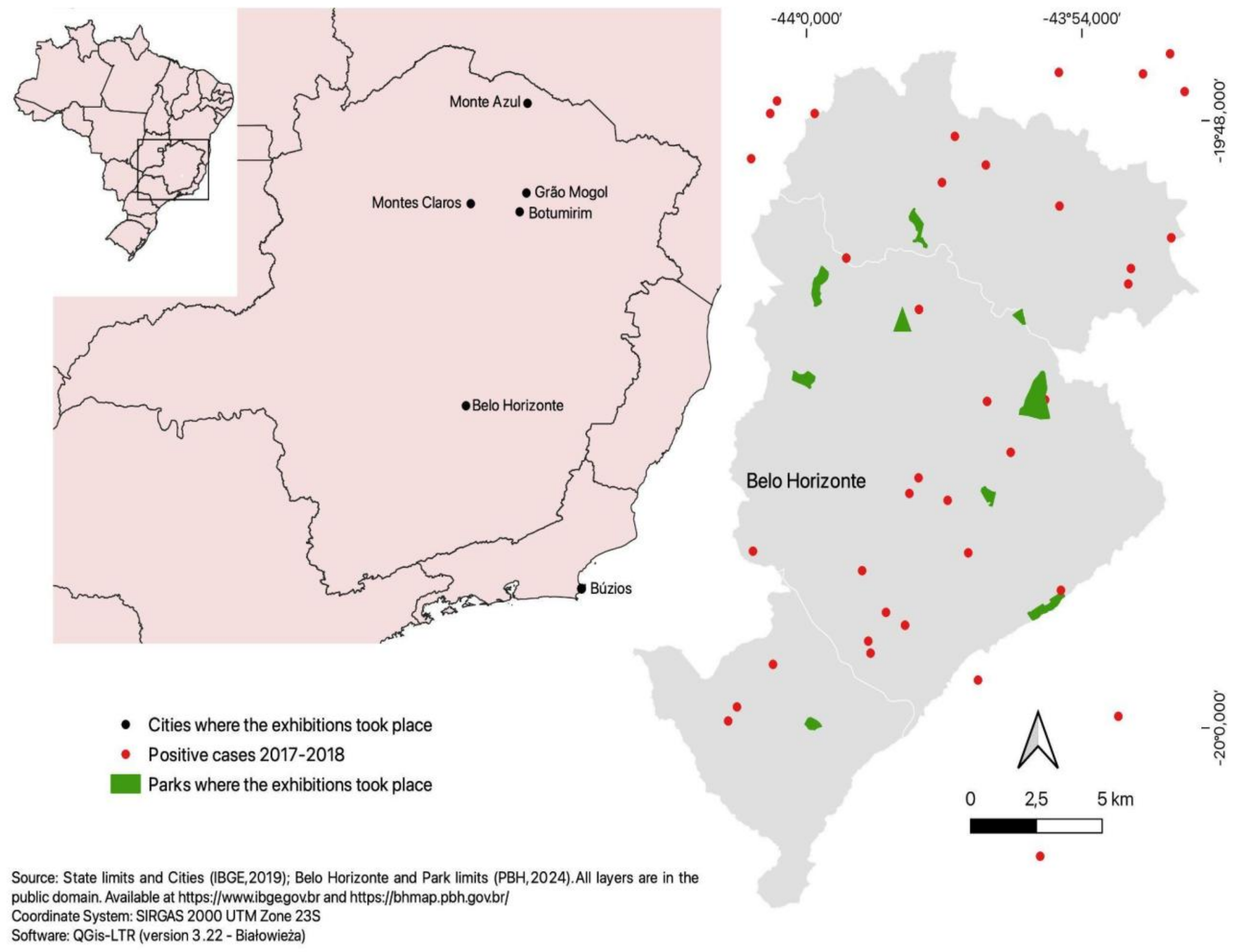

Fig A: **Cities Where "CREATE and CONNECT" Exhibition Took Place in 2023 and 2024, Brazil.** From left to right: map of Brazil; part of Southeast region showing municipalities where the exhibition took place. Map of Belo Horizonte: red dots show places where carcasses of non-human primate infected with yellow fever virus were collected during outbreaks in 2017 and 2018 and green areas represent the parks where the exhibition took place in Belo Horizonte. The maps were created with the software: QGis-LTR (version 3 .22 - Białowieża), all layers are in the public domain available from https://www.ibge.gov.br, and https://bhmap.pbh.gov.br/. Coordinate System: SIRGAS 2000 UTM Zone 23S.

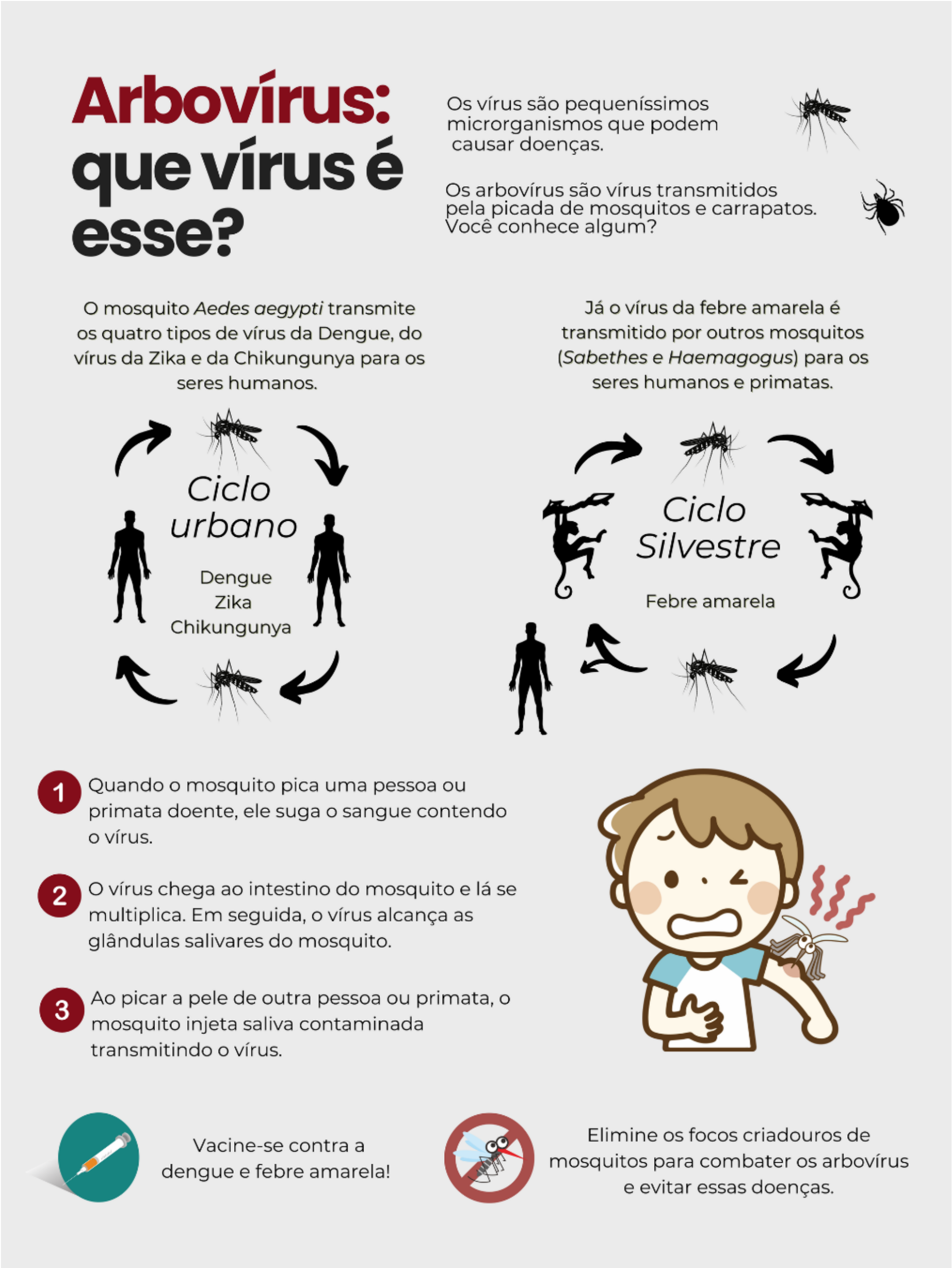

Fig B: Arbovirus transmission. Poster (written in Portuguese), used to display basic information on urban and sylvatic cycles of arboviruses, how arboviruses infect mosquito and are transmitted. The poster was printed on PVC-coated canvas, allowing them to be reused. The poster was created using freely available images sourced from the internet under open-access terms (https://openclipart.org/detail/334764/bug-bites-a-kid; https://openclipart.org/detail/279073/simple-tick-ixodes-ricinus-silhouette#google_vignette; https://openclipart.org/detail/305715/mosquito; https://openclipart.org/detail/328344/circle-slash-no-symbol; https://openclipart.org/detail/327908/syringe-in-a-circle; https://openclipart.org/detail/182185/man-shape; https://openclipart.org/detail/7547/monkey-silhouette; https://commons.wikimedia.org/wiki/File:Curved_solid_arrow.svg; https://openclipart.org/detail/315188/mosquito-5) [Supplementary references 1-9].

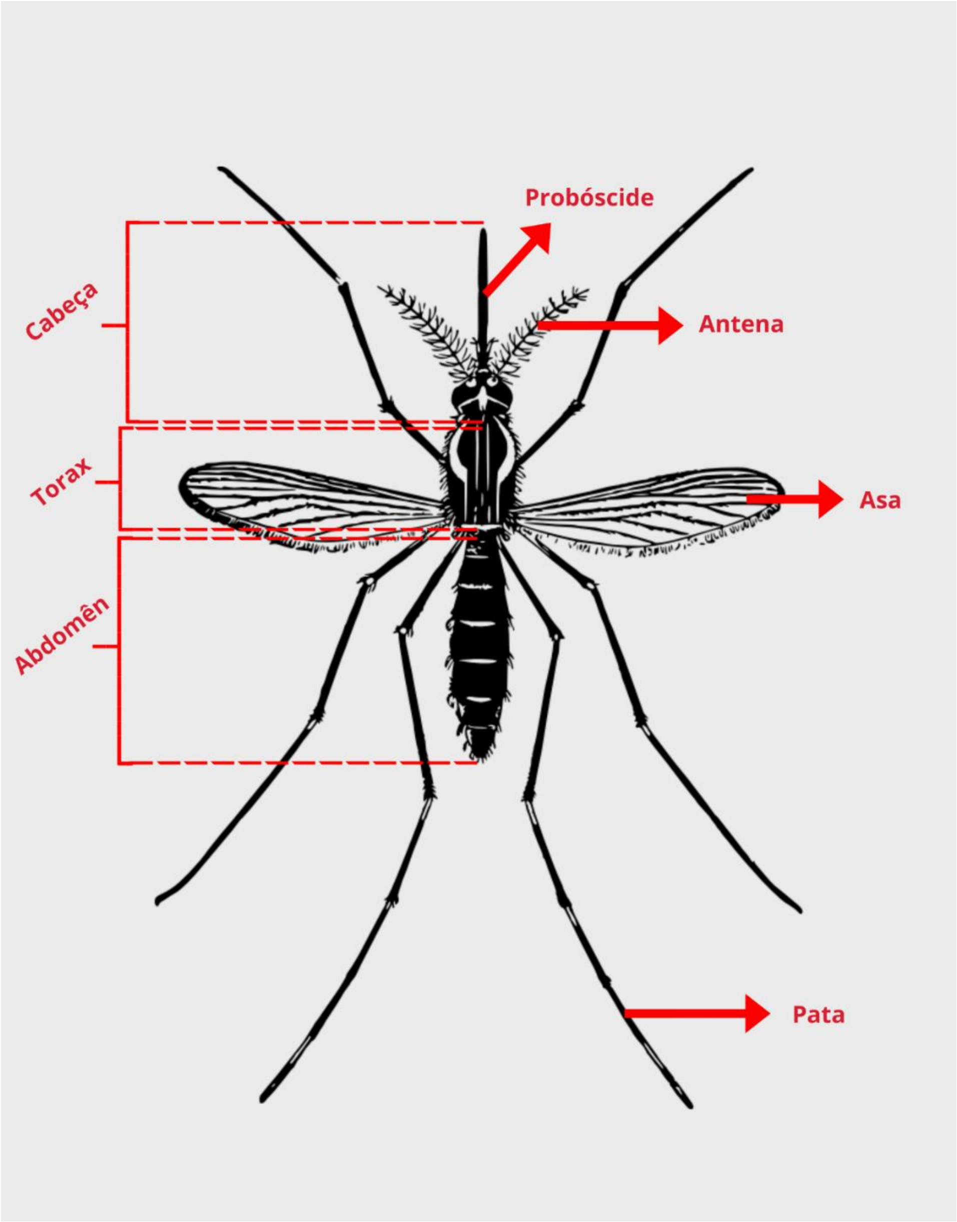

Fig C: Mosquitoes anatomy sketch. Schematic representation of the mosquito's external anatomy, highlighting the main body regions (written in Portuguese). This figure was created using freely available images sourced from the internet under open-access terms (https://openclipart.org/detail/269837/mosquito-2;https://commons.wikimedia.org/wiki/File:Red_arrow_southeast.svg) [Supplementary references 10-11].

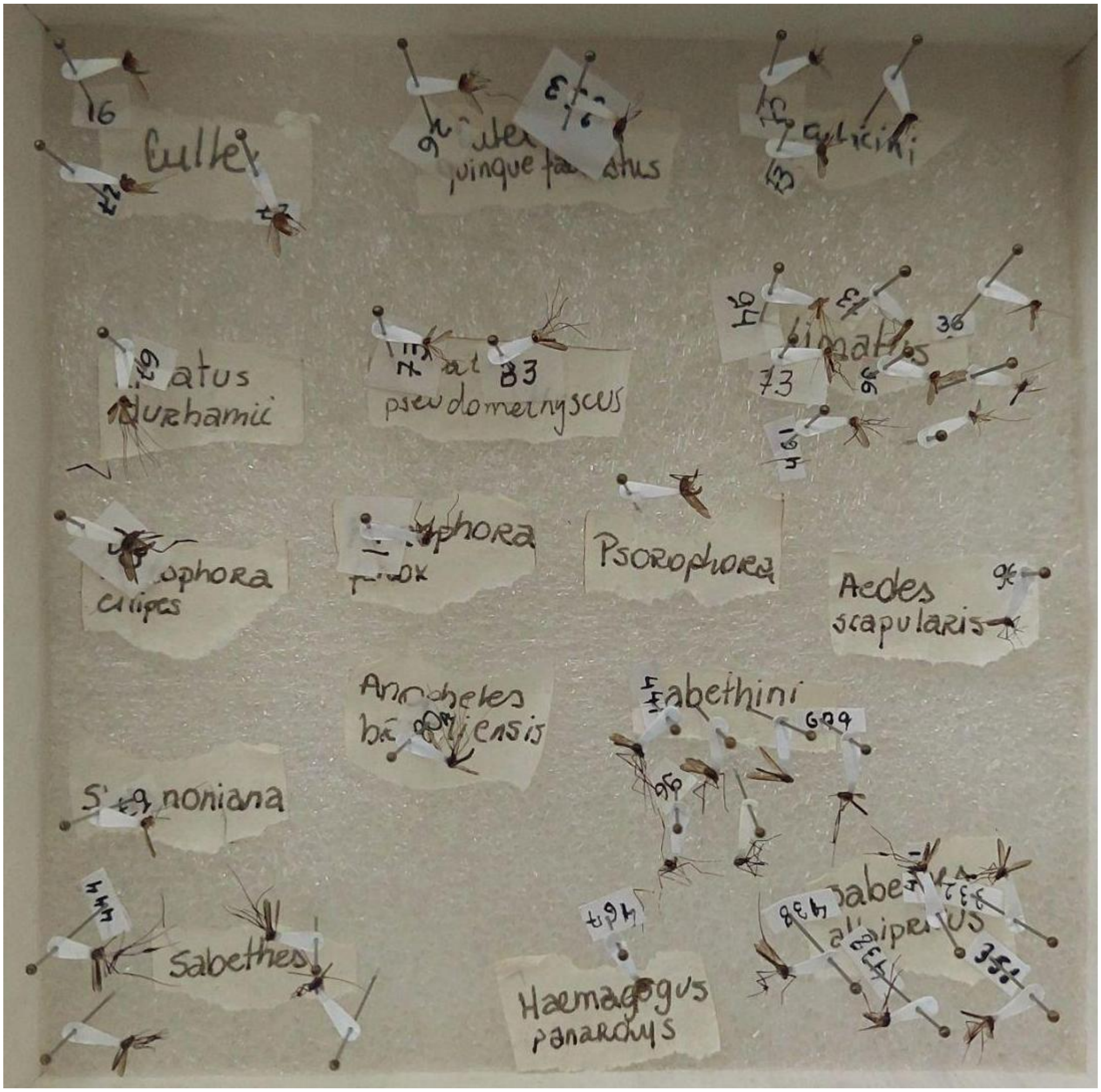

Fig D: Entomological collection of mosquito vectors of arboviruses. Preserved mosquitoes were exhibited for visitors under hand magnifying glasses or steromicroscope.

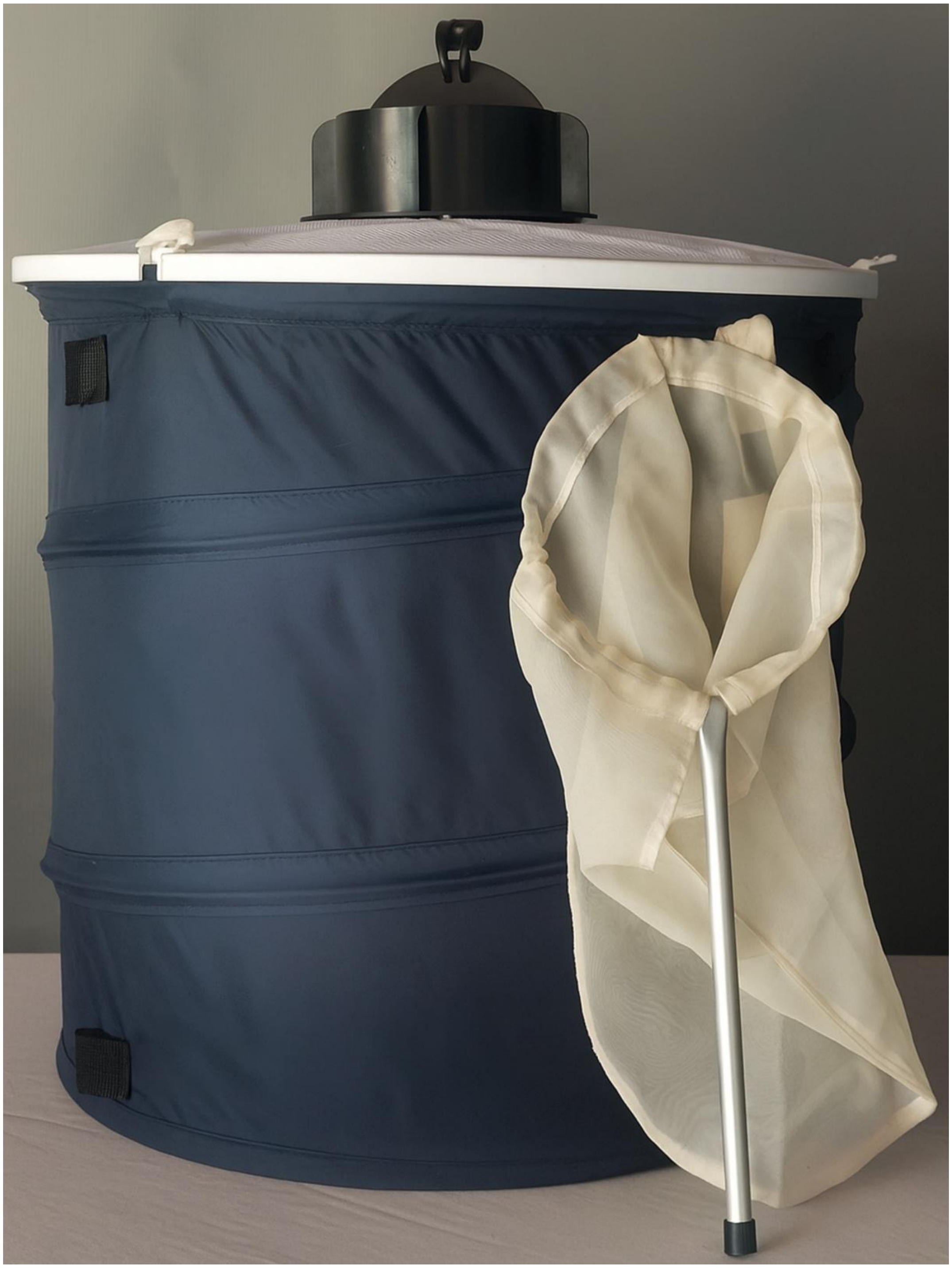

Fig E. Trap and hand nets for mosquito sampling. The equipment was used to illustrate how mosquitoes are collected during the entomological research in field conditions.

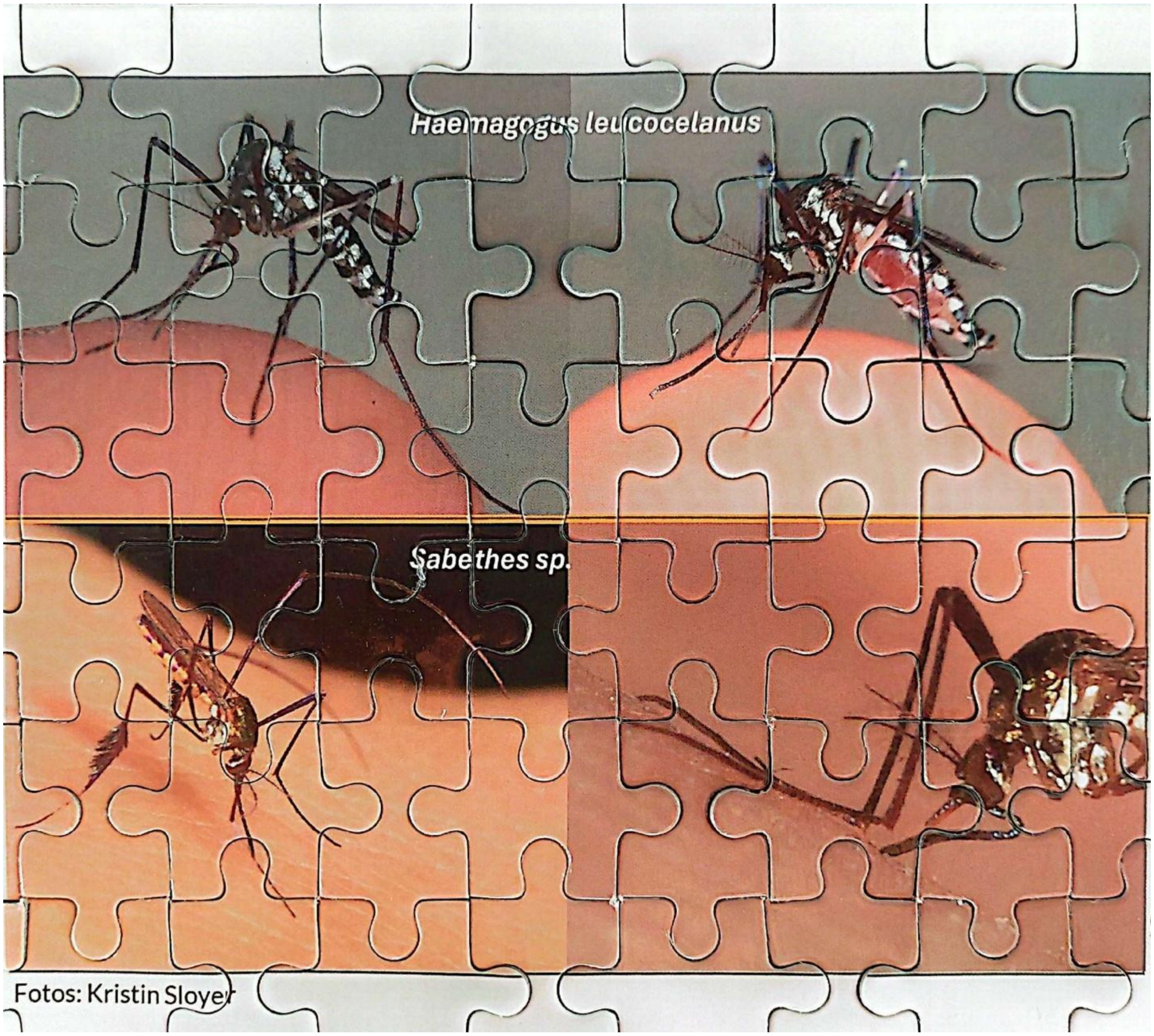

Fig F: Mosquito puzzle. In this game, the player looks at the picture or reference, finds and connects matching pieces by their shapes and colors, and continues until the entire image is complete.

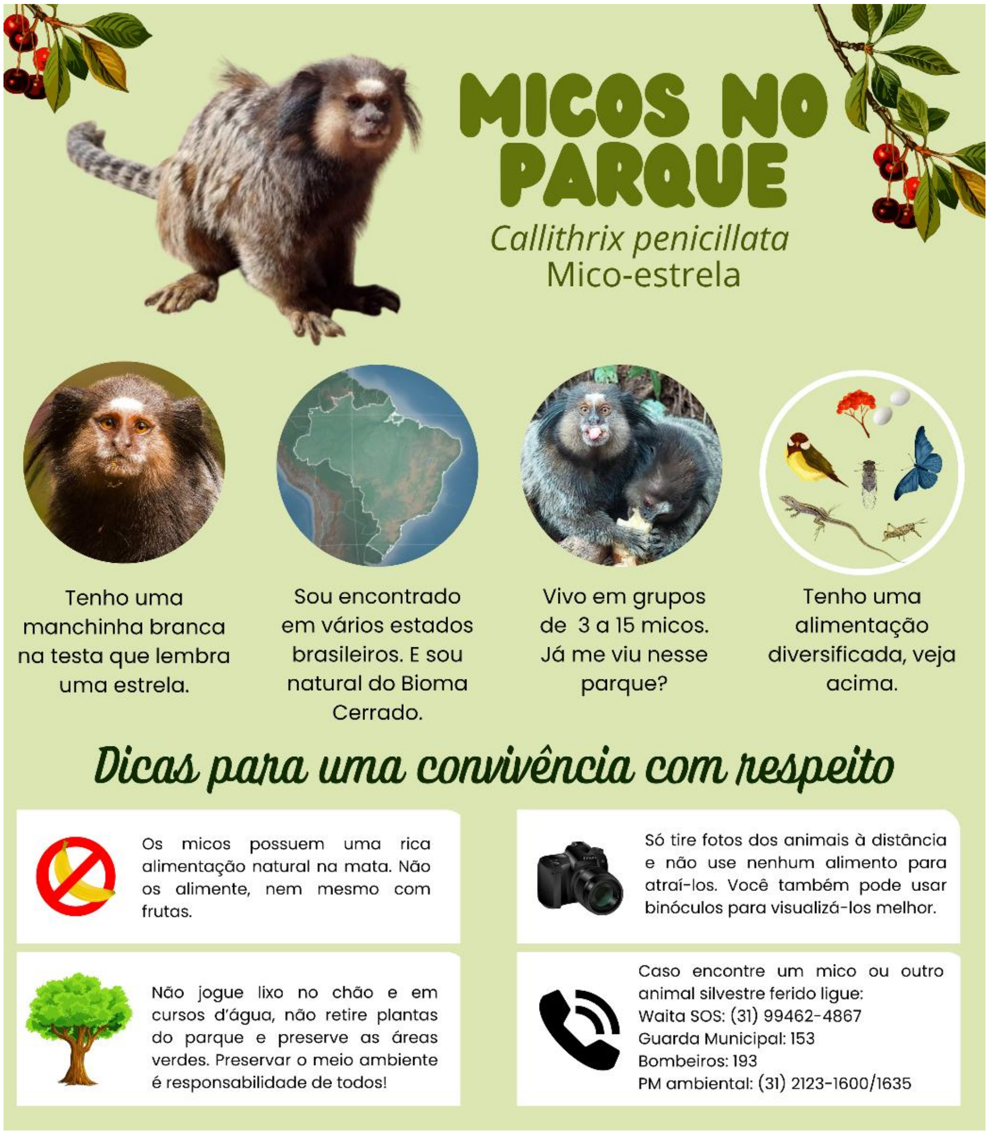

Fig G: Black-tufted-marmoset poster. Poster (written in Portuguese) was used to display basic information about the biology and ecology of the species. The poster also included information about the safe coexistence and observation of wild animals and basic guidelines in case an injured or weakened animal is found in the parks. The poster was printed on PVC-coated canvas, allowing them to be reused. The poster was created using freely available images sourced from the internet under open-access terms (https://openclipart.org/detail/240979/sour-cherry-tree-2-low-resolution; https://openclipart.org/detail/327533/grasshopper-2-isolated; https://openclipart.org/detail/168562/camera-no-filters; https://openclipart.org/detail/263892/colorful-natural-tree; https://openclipart.org/detail/304028/cuban-ameiva; https://openclipart.org/detail/229578/autumn2seamless-pattern; https://commons.wikimedia.org/wiki/File:Brazil_w2_locator.svg; https://openclipart.org/detail/266382/cicada; https://openclipart.org/detail/313294/phone; https://openclipart.org/detail/181696/blue-butterfly; https://openclipart.org/detail/84217/banana; https://openclipart.org/detail/328344/circle-slash-no-symbol) [Supplementary references 7,12-22].

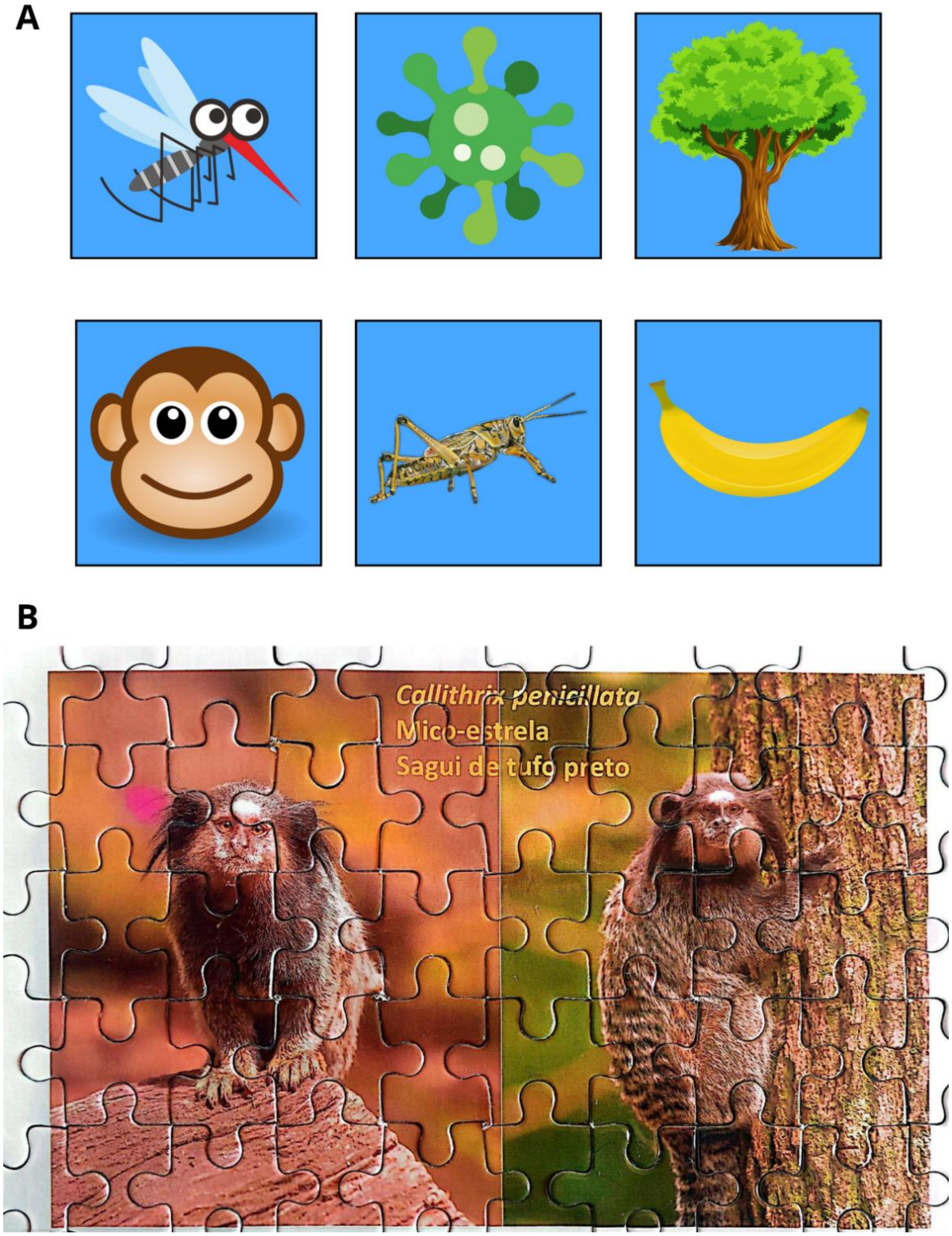

Fig H: Memory game (A) and Marmoset puzzle (B). A: In the memory cards are shuffled and placed face down in a grid. Players take turns flipping over two cards at a time. The game continues until all pairs are found, and the player with the most pairs wins. **B:** Black-tufted-marmoset (*Callithrix penicillata*) puzzle. Games were printed and laminated, allowing them to be reused. A combination of original photographs and freely available images sourced from the internet under open-access terms were used (https://openclipart.org/detail/327533/grasshopper-2-isolated; https://openclipart.org/detail/84217/banana; https://openclipart.org/detail/289605/virus; https://openclipart.org/detail/263892/colorful-natural-tree; https://openclipart.org/detail/81865/funny-monkey-face; https://openclipart.org/detail/315188/mosquito-5) [Supplementary references 8,18-19,21,23-24].

Supplementary references:

Some figures were created using images licensed under the Creative Commons Attribution 4.0 International License (CC BY 4.0):

1 openclipart.org [Internet]. Life cycle of the mosquito; c2020 [cited 2025 Jul 30]. Available from: https://openclipart.org/detail/324257/life-cycle-of-the-mosquito.

2 openclipart.org [Internet]. Simple tick Ixodes ricinus silhouette; c2017 [cited 2025 Jul 30]. Available from: https://openclipart.org/detail/279073/simple-tick-ixodes-ricinus-silhouette.

3 openclipart.org [Internet]. Man shape; c2013 [cited 2025 Jul 30]. Available from: https://openclipart.org/detail/182185/man-shape.

4 openclipart.org [Internet]. Monkey silhouette; c2007 [cited 2025 Jul 30]. Available from: https://openclipart.org/detail/7547/monkey-silhouette.

5 openclipart.org [Internet]. Bug bites a kid; c2021 [cited 2025 Jul 30]. Available from: https://openclipart.org/detail/334764/bug-bites-a-kid.

6 openclipart.org [Internet]. Syringe in a circle; c2021 [cited 2025 Jul 30]. Available from: https://openclipart.org/detail/327908/syringe-in-a-circle.

7 openclipart.org [Internet]. Circle slash no symbol; c2021 [cited 2025 Jul 30]. Available from: https://openclipart.org/detail/328344/circle-slash-no-symbol.

8 openclipart.org [Internet]. Mosquito 5; c2019 [cited 2025 Jul 30]. Available from: https://openclipart.org/detail/315188/mosquito-5.

9 commons.wikimedia.org [Internet]. Curved solid arrow; c2022 [cited 2025 Jul 30]. Available from: https://commons.wikimedia.org/wiki/File:Curved_solid_arrow.svg.

10 openclipart.org [Internet]. Mosquito 2; c2016 [cited 2025 Jul 30]. Available from: https://openclipart.org/detail/269837/mosquito-2.

11 commons.wikimedia.org [Internet]. Red arrow southeast; c2018 [cited 2025 Jul 30]. Available from: https://commons.wikimedia.org/wiki/File:Red_arrow_southeast.svg.

12 commons.wikimedia.org [Internet] Brazil w2 locator; c2022 [cited 2025 Jul 30]. Available from: https://commons.wikimedia.org/wiki/File:Brazil_w2_locator.svg.

13 openclipart.org [Internet]. Sour cherry tree 2 (low resolution); c2016 [cited 2025 Jul 30]. Available from: https://openclipart.org/detail/240979/sour-cherry-tree-2-low-resolution.

14 openclipart.org [Internet]. Autumn2seamless pattern; c2015 [cited 2025 Jul 30]. Available from: https://openclipart.org/detail/229578/autumn2seamless-pattern.

15 openclipart.org [Internet]. Cicada; c2016 [cited 2025 Jul 30]. Available from: https://openclipart.org/detail/266382/cicada.

16 openclipart.org [Internet]. Cuban Ameiva; c2018 [cited 2025 Jul 30]. Available from: https://openclipart.org/detail/304028/cuban-ameiva.

17 openclipart.org [Internet]. Blue butterfly; c2013 [cited 2025 Jul 30]. Available from: https://openclipart.org/detail/181696/blue-butterfly.

18 openclipart.org [Internet]. Grasshopper 2 isolated; c2021 [cited 2025 Jul 30]. Available from: https://openclipart.org/detail/327533/grasshopper-2-isolated.

19 openclipart.org [Internet]. Banana; c2010 [cited 2025 Jul 30]. Available from: https://openclipart.org/detail/84217/banana.

20 openclipart.org [Internet]. Camera no filters; c2012 [cited 2025 Jul 30]. Available from: https://openclipart.org/detail/168562/camera-no-filters.
21 openclipart.org [Internet]. Colorful natural tree; c2016 [cited 2025 Jul 30]. Available from: https://openclipart.org/detail/263892/colorful-natural-tree.
22 openclipart.org [Internet]. Phone; c2019 [cited 2025 Jul 30]. Available from: https://openclipart.org/detail/313294/phone.
23 openclipart.org [Internet]. Virus; c2017 [cited 2025 Jul 30]. Available from: https://openclipart.org/detail/289605/virus.
24 openclipart.org [Internet]. Funny monkey face; c2010 [cited 2025 Jul 30]. Available from: https://openclipart.org/detail/81865/funny-monkey-face.
