# Supplementary material for: CREATE and CONNECT: Arboviruses at the intersection of research and community outreach
Source: PLoS Negl Trop Dis. 2025 Aug 22;19(8):e0013436. doi: 10.1371/journal.pntd.0013436 (PMC12483539; doi:10.1371/journal.pntd.0013436)
Supplement: S2 File — (S2_File.PDF) [file pntd.0013436.s002.pdf]

S2 File - Folder distributed to visitors and Educational Book.

CREATE and CONNECT - Arboviruses at the intersection of Research and Community Outreach –

Jacob et al 2025.

Sapajus sp.

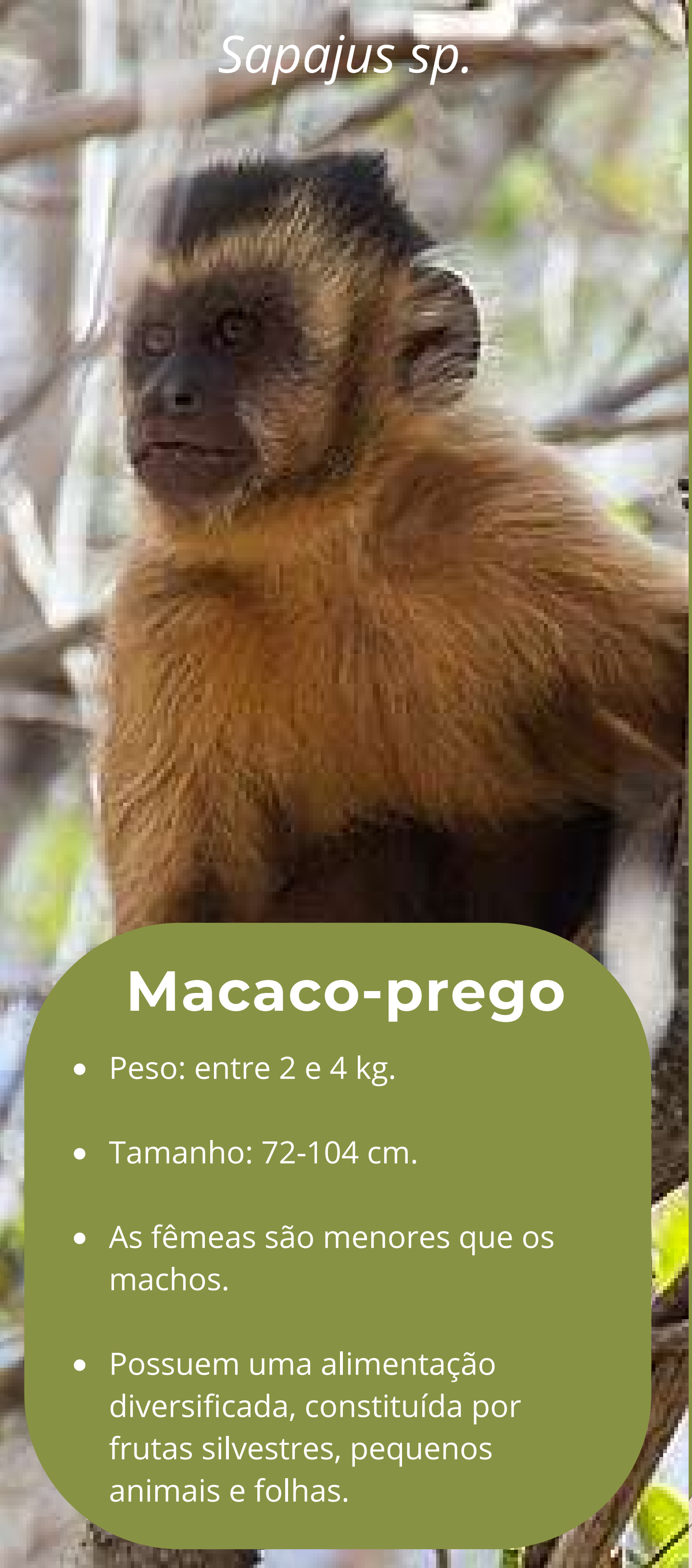

Macaco-prego

- Peso: entre 2 e 4 kg.
- Tamanho: 72-104 cm.
- As fêmeas são menores que os machos.
- Possuem uma alimentação diversificada, constituída por frutas silvestres, pequenos animais e folhas.

Bem-vindos

Somos cientistas do grupo de pesquisa CREATE-NEO e estudamos a saúde dos primatas não humanos, nos parques de Belo Horizonte.

Este guia foi criado para fornecer informações sobre as duas espécies de primatas encontrados nos parques de Belo Horizonte, bem pertinho de nossas casas: o mico-estrela (*Callithrix penicillata*) e o macaco prego (*Sapajus nigritus*).

Vamos explorar sua história natural, comportamento, habitat, ameaças e medidas de conservação. Aprenda mais sobre eles e se torne também um **Guardião dos Primatas!**

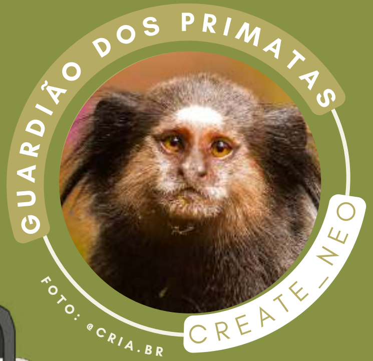

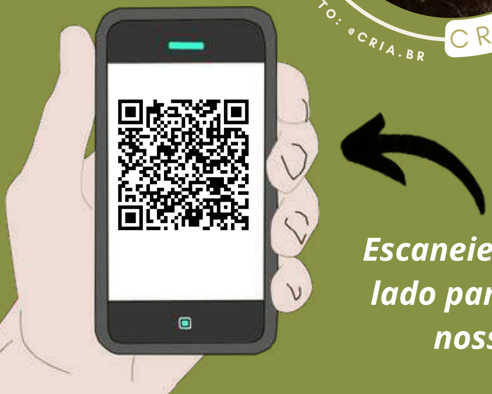

Escaneie o código ao lado para acessar o nosso guia.

Callithrix penicillata

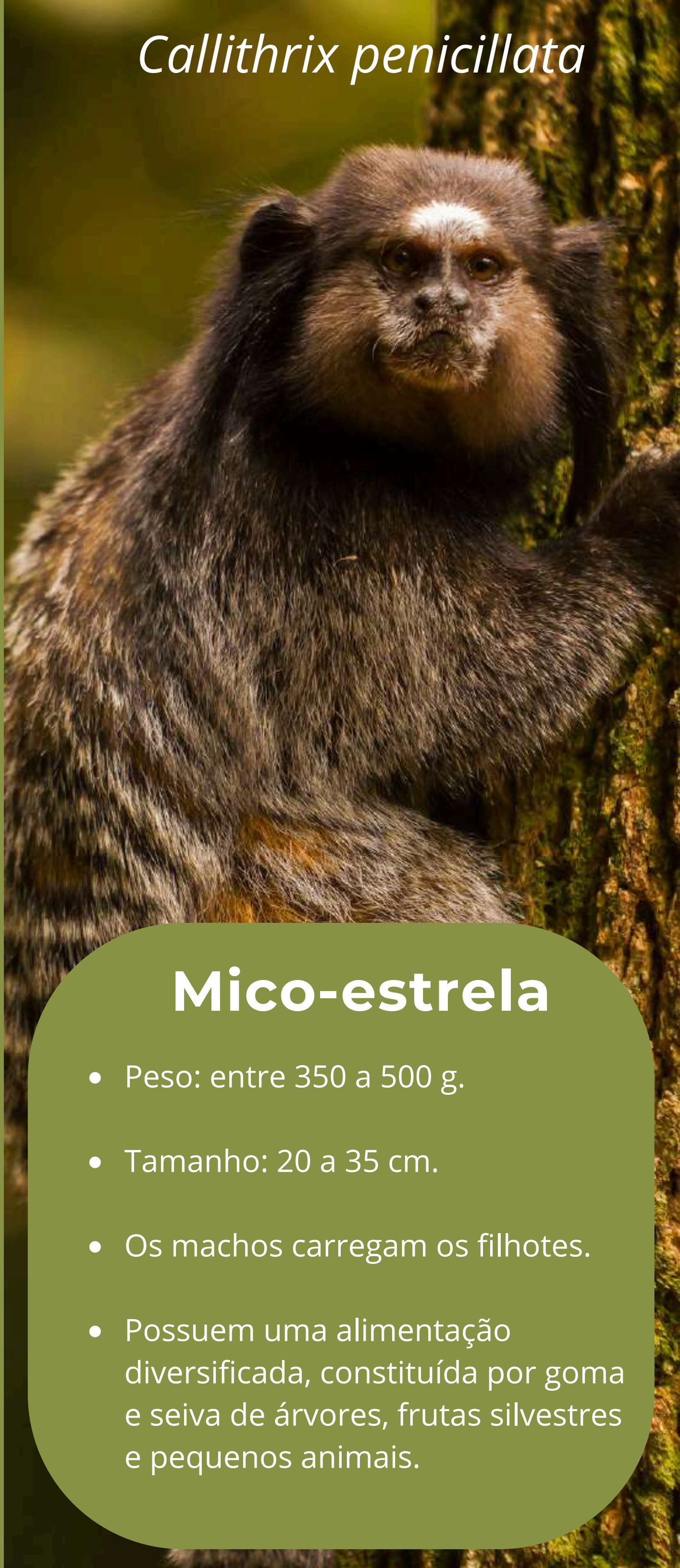

Mico-estrela

- Peso: entre 350 a 500 g.
- Tamanho: 20 a 35 cm.
- Os machos carregam os filhotes.
- Possuem uma alimentação diversificada, constituída por goma e seiva de árvores, frutas silvestres e pequenos animais.

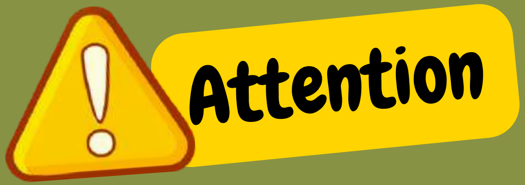

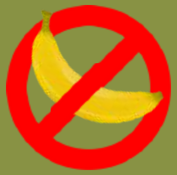

Os micos possuem uma rica alimentação natural na mata. Não os alimente, nem mesmo com frutas.

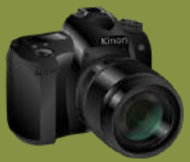

Só tire fotos dos animais à distância e não use nenhum alimento para atraí-los. Você também pode usar binóculos para visualizá-los melhor.

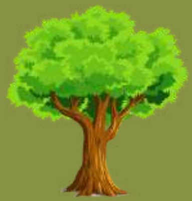

Não jogue lixo no chão e em cursos d'água, não retire plantas do parque e preserve as áreas verdes.

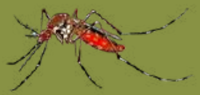

Os primatas não transmitem o vírus da febre amarela, e sim, os mosquitos. Elimine focos criadouros de mosquitos e vacine-se contra febre amarela e dengue.

Achou um animal silvestre ferido? Não toque!

Entre em contato imediatamente com uma das instituições abaixo:

Waita SOS animais silvestres
(31) 99462-4867

Guarda Municipal
153

Bombeiros
193

PM Ambiental
(31) 2123-1600/1635

Elaboração

Marcelle Oliveira; Mikaelly Frasson
Daniel Jacob; Ana Maria Paschoal; Daniel Vilela; Betânia Drumond.

Imagens usadas

AEste guia foi elaborado utilizando uma combinação de fotografias (de Eric Souz) e imagens disponíveis gratuitamente na Internet em termos de acesso livre ao abrigo da licença CC BY 4.0.

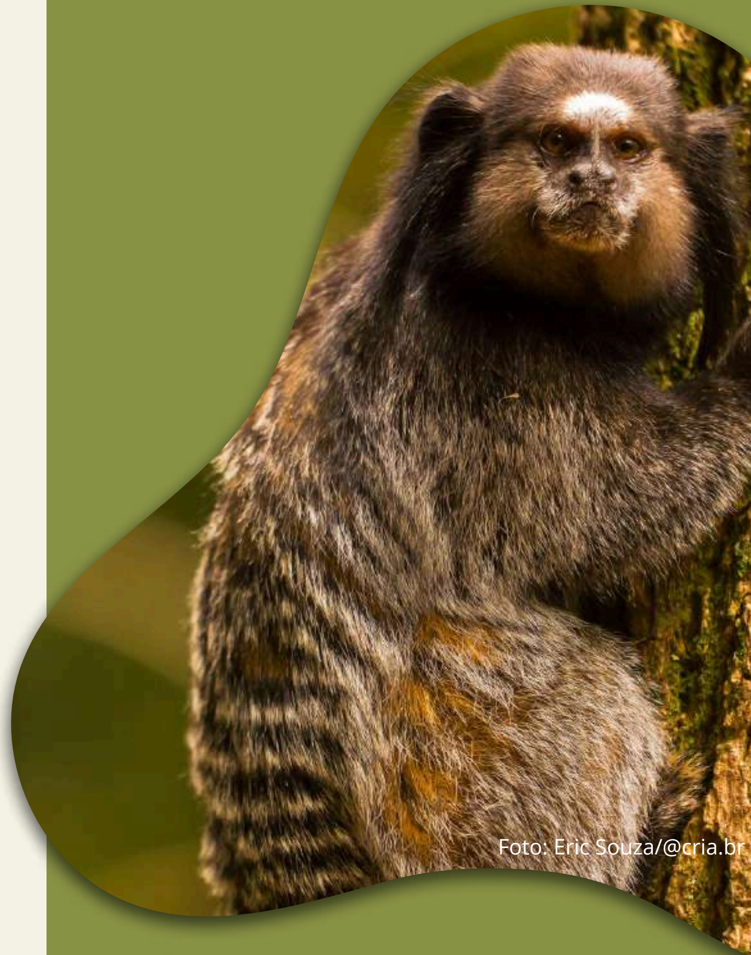

Foto: Eric Souza/@cria.br

Explore e Conheça

O incrível mundo dos primatas nos parques urbanos de Belo Horizonte

Sapajus sp.

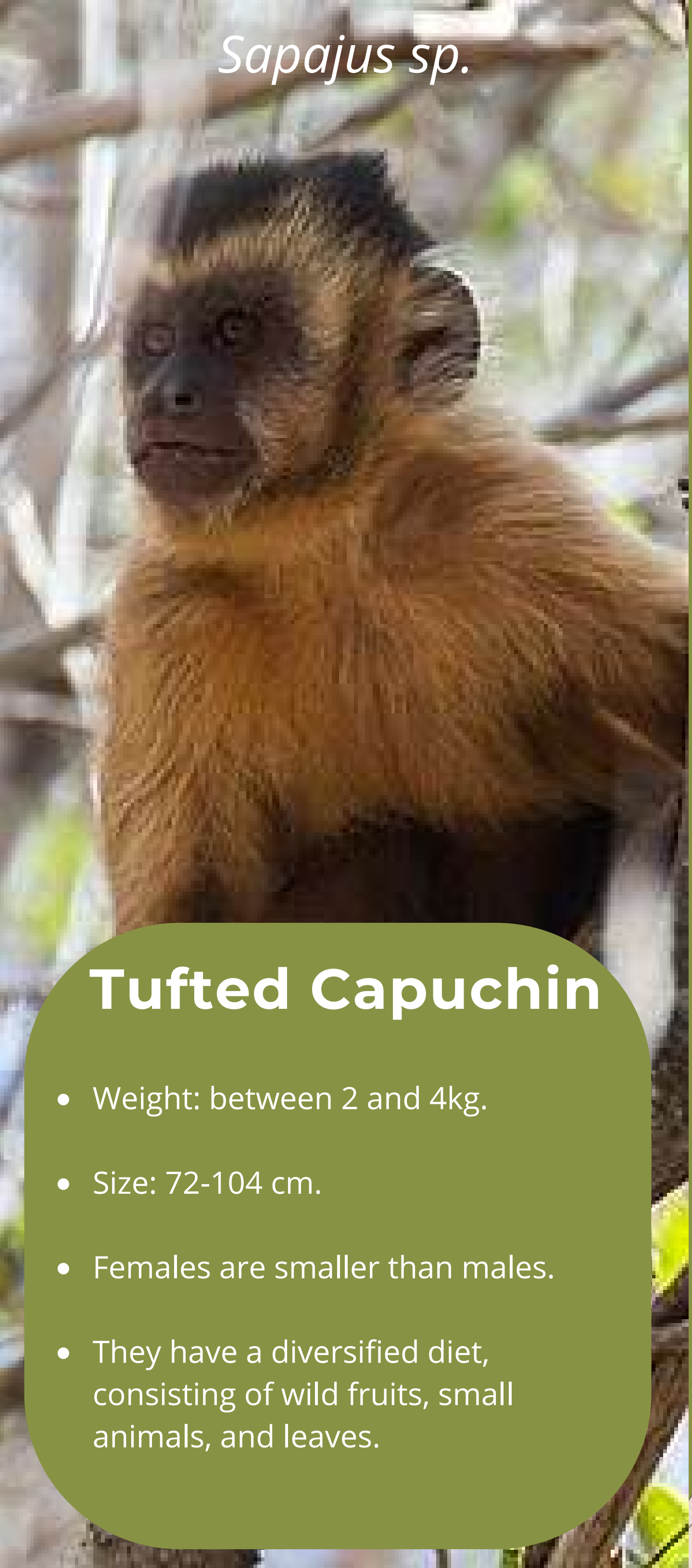

Tufted Capuchin

- Weight: between 2 and 4kg.
- Size: 72-104 cm.
- Females are smaller than males.
- They have a diversified diet, consisting of wild fruits, small animals, and leaves.

Welcome

We are scientists from the CREATE-NEO research group, and we study the health of non-human primates in the parks of Belo Horizonte. This guide was created to provide information about the two species of non-human primates observed close to our homes, in urban parks of Belo Horizonte: the black-tufted marmoset (*Callithrix penicillata*) and the tufted capuchin (*Sapajus nigritus*).

Let's explore their natural history, behavior, habitat, and how to preserve them. Come and learn about them and become a **Guardian of Primates!**

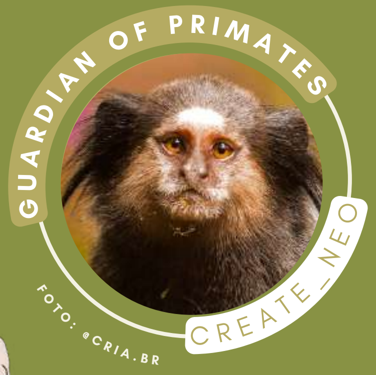

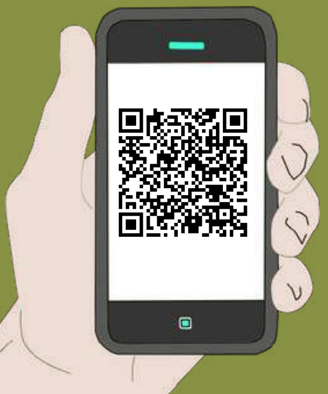

Scan the code to access our guide.

Callithrix penicillata

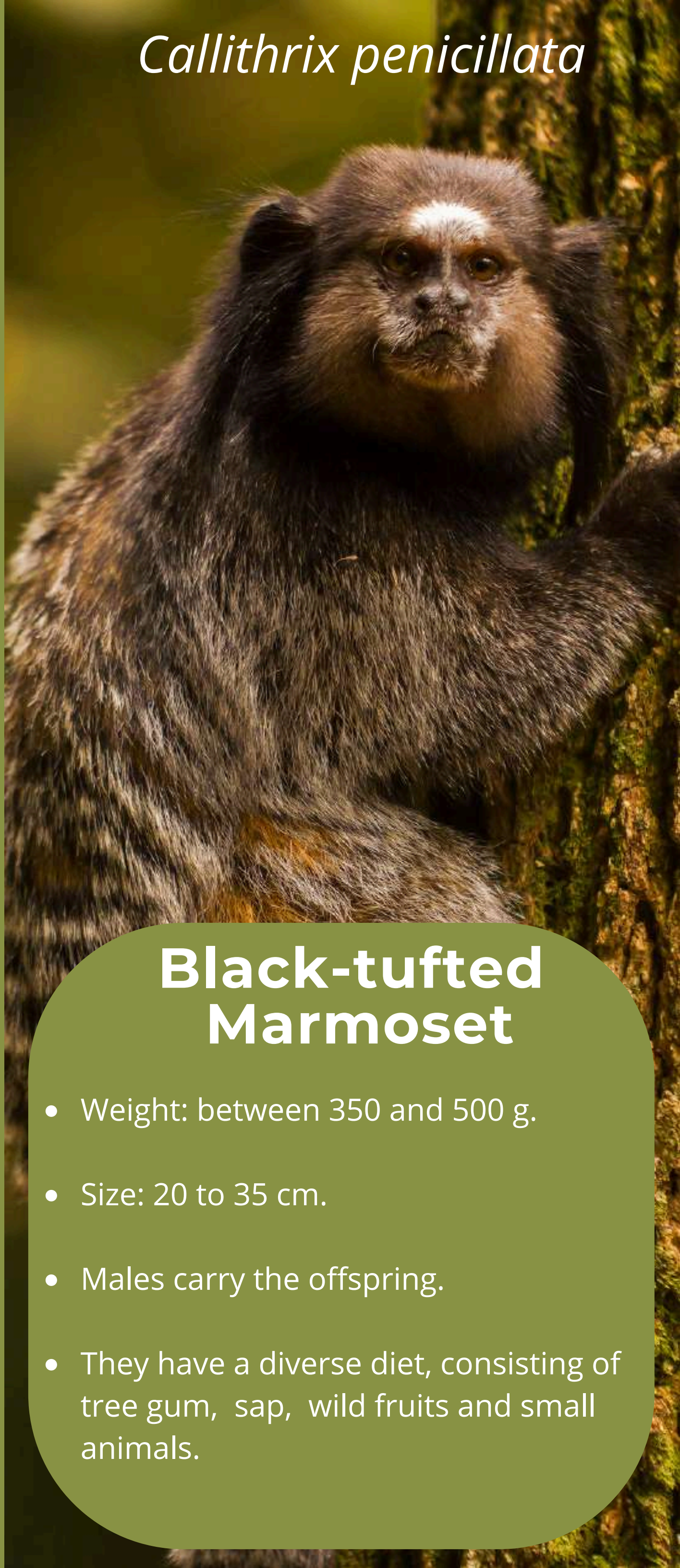

Black-tufted Marmoset

- Weight: between 350 and 500 g.
- Size: 20 to 35 cm.
- Males carry the offspring.
- They have a diverse diet, consisting of tree gum, sap, wild fruits and small animals.

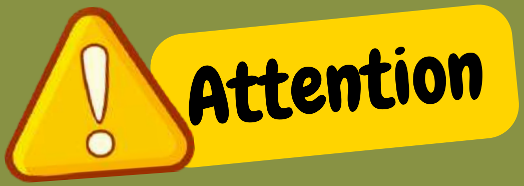

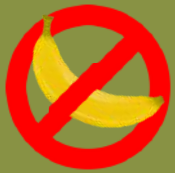

Marmosets have a rich natural diet in the forest. Do not feed them, not even with fruits.

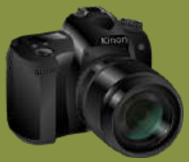

Only take photos of the animals from a safe distance and do not use any food to lure them. You can also use binoculars to view them better.

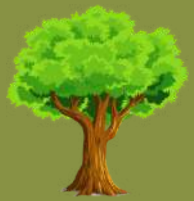

Do not litter on the ground, in lakes or water courses. Do not remove plants from the park, and preserve green areas.

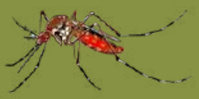

Primates do not transmit yellow fever virus. Yellow fever virus is transmitted by mosquitoes. Eliminate mosquito breeding grounds and get vaccinated against yellow fever and dengue.

Did you find an injured wild animal? Do not touch the animal.

Immediately contact a responsible institution:

Waita SOS Wildlife
(31) 99462-4867

Municipal guard
153

Firefighters
193

Environmental Police
(31) 2123-1600/1635

Elaboration

Marcelle Oliveira; Mikaelly Frasson
Daniel Jacob; Ana Maria Paschoal; Daniel Vilela; Betânia Drumond.

Images used

This guide was created using a combination of photographs (by Eric Souz) and freely available images from the Internet under open-access terms in accordance with the CC BY 4.0 license.

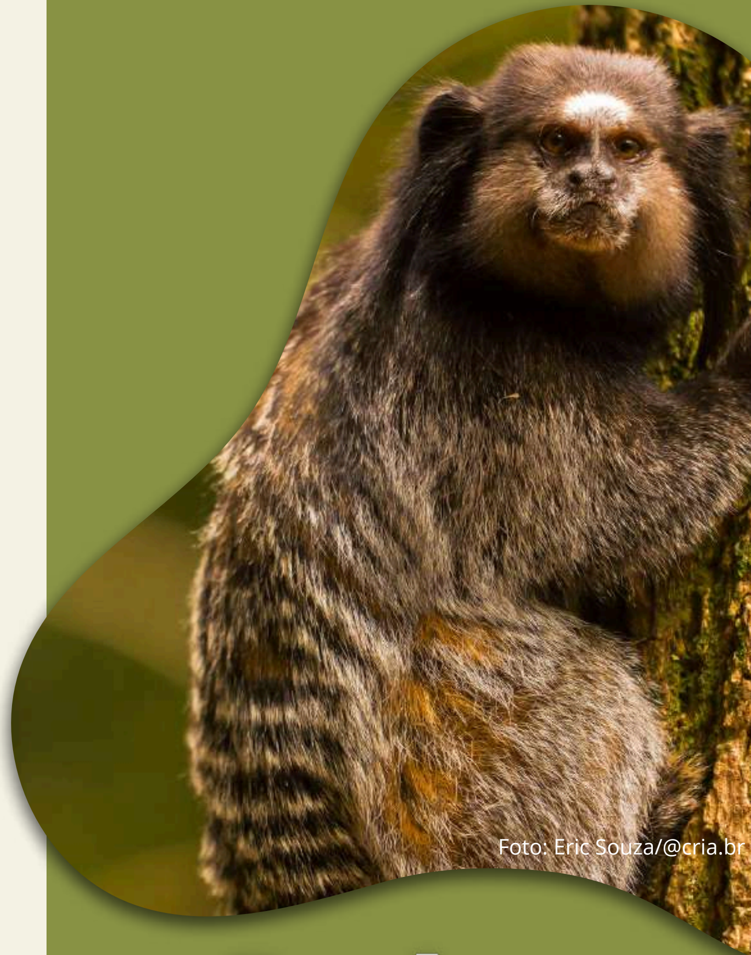

Foto: Eric Souza/@cria.br

Explore and Discover

The Amazing World of Primates in Urban Parks of Belo Horizonte

Explore e Conheça

O incrível mundo dos Primatas nos parques de Belo Horizonte

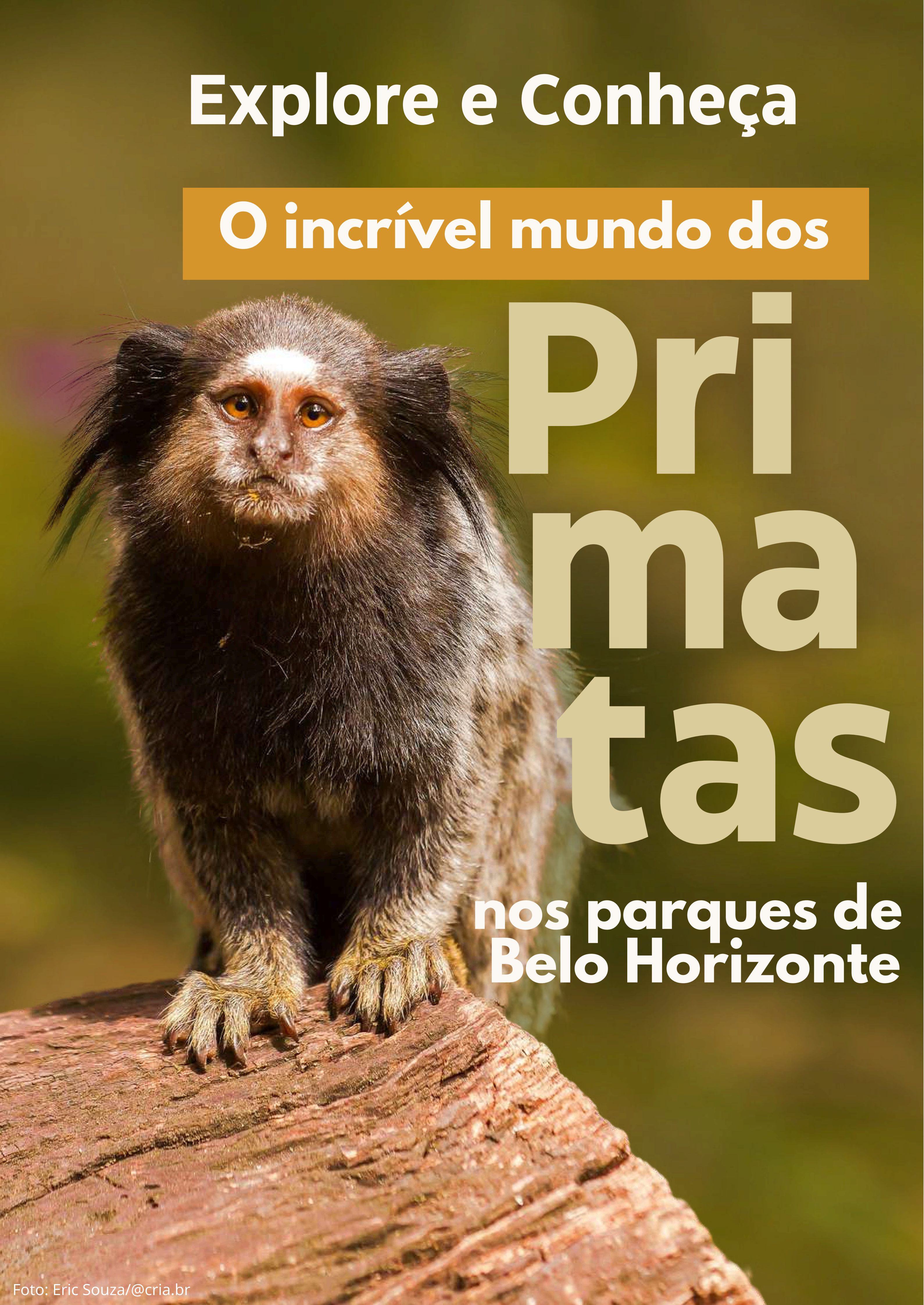

Foto: Eric Souza/@cria.br

Prefácio

Bem-vindos ao guia educacional **"O incrível mundo dos primatas nos Parques Urbanos de Belo Horizonte"**.

Este guia foi criado para fornecer informações importantes sobre duas espécies fascinantes de primatas encontradas em Belo Horizonte: o mico estrela (*Callithrix penicillata*) e o macaco prego (*Sapajus nigritus*).

Vamos explorar curiosidades sobre a biologia, ecologia e comportamentos destes animais. Também vamos abordar as principais ameaças e condutas de boas práticas para convivermos com essas espécies de forma respeitosa. Esperamos que assim, você se apaixone pelos primatas assim como nós.

Mikaelly Frasson Biccas
Marcelle Alves de Oliveira
Daniel Jacob Circuncisão
Ana Maria de Oliveira Paschoal
Daniel Ambrósio Vilela
Betânia Paiva Drumond

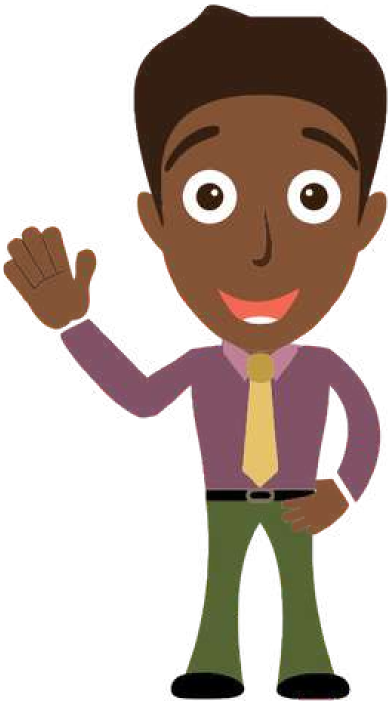

Os parques urbanos
de Belo Horizonte

A cidade de Belo Horizonte se destaca por possuir mais de 1070 hectares de áreas verdes urbanas, totalizando aproximadamente 73 parques distribuídos entre as 8 regionais da cidade. Além disso, a cidade possui diversas áreas verdes como jardim botânico, zoológico e centros de vivência agroecológica.

Em Belo Horizonte encontramos espécies de plantas e animais dos biomas Cerrado e Mata Atlântica. A diversidade é surpreendente: mais de 200 espécies de animais habitam esses espaços, junto a mais de 1000 espécies vegetais e inúmeras nascentes.

Com o avanço da urbanização, muitos desses parques encontram-se em proximidade direta com áreas residenciais e comerciais. Assim, os animais silvestres podem se aproximar das pessoas, o que pode ser encantador, mas também pode trazer riscos para as pessoas e animais.

Neste breve guia, você será convidado a explorar o fascinante universo dos primatas que habitam os parques urbanos de Belo Horizonte e descobrirá como é possível conviver com essas espécies de maneira harmoniosa e respeitosa.

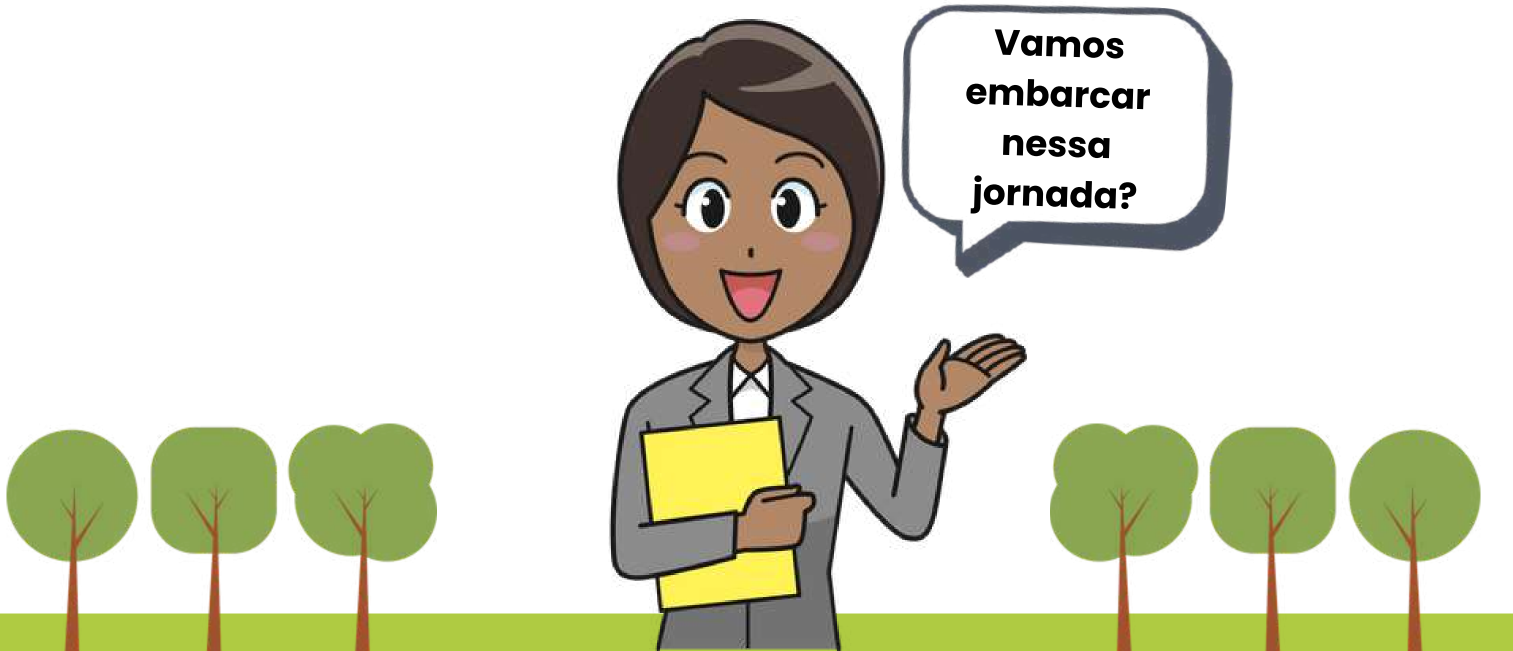

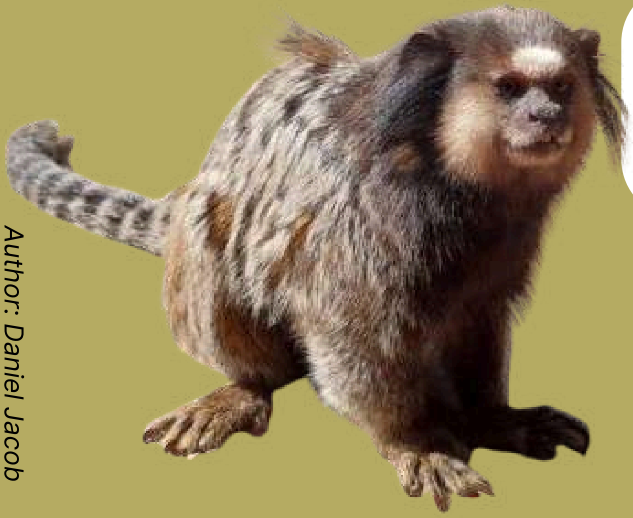

Author: Daniel Jacob

MICO-ESTRELA

Callithrix penicillata

COMO EU SOU

- Tenho até 60 cm de tamanho (corpo + cauda).
- Peso de 350 a 500 gramas.
- Sou ágil e sociável: vivo em grupos de 3 a 15 animais.

POR QUE ESSE NOME?

Eu sou o mico-estrela. Tenho esse nome por causa da mancha branca em forma de estrela na minha testa. Sabia que também sou chamado de sagui- de-tufo-preto?

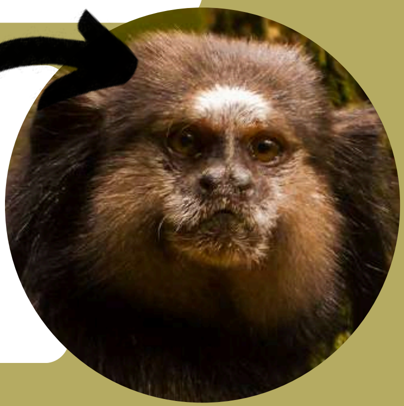

Author: Eric Souza

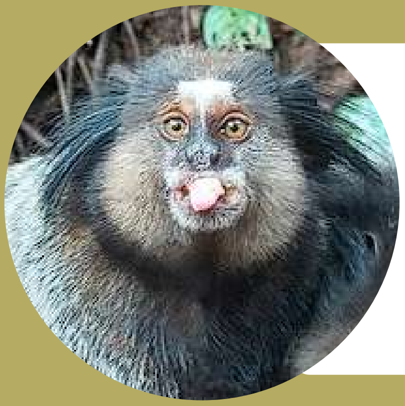

Author: Daniel Jacob

NÃO É DIFÍCIL ME ENCONTRAR

Sou muito adaptável, provavelmente você já me viu em muitos lugares da nossa cidade. Na minha família minha mãe sempre tem gêmeos, então, eu tenho muitos irmãos!

ALIMENTAÇÃO

Eu tenho muito apetite! Eu gosto de comer de tudo: insetos, pequenos vertebrados e invertebrados, frutos silvestres e goma das árvores.

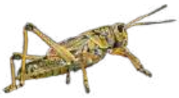

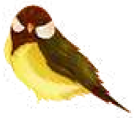

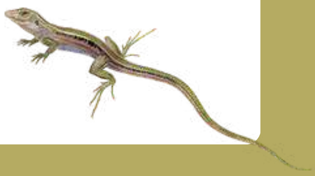

Na mata eu não passo fome. Por isso, eu não preciso que você me ofereça sua comida! A sua comida não me faz muito bem e daqui a pouco contarei porque. Mas antes, vocês irão conhecer meus amigos macacos-pregos.

MONO CAPUCHINO

Sapajus sp.

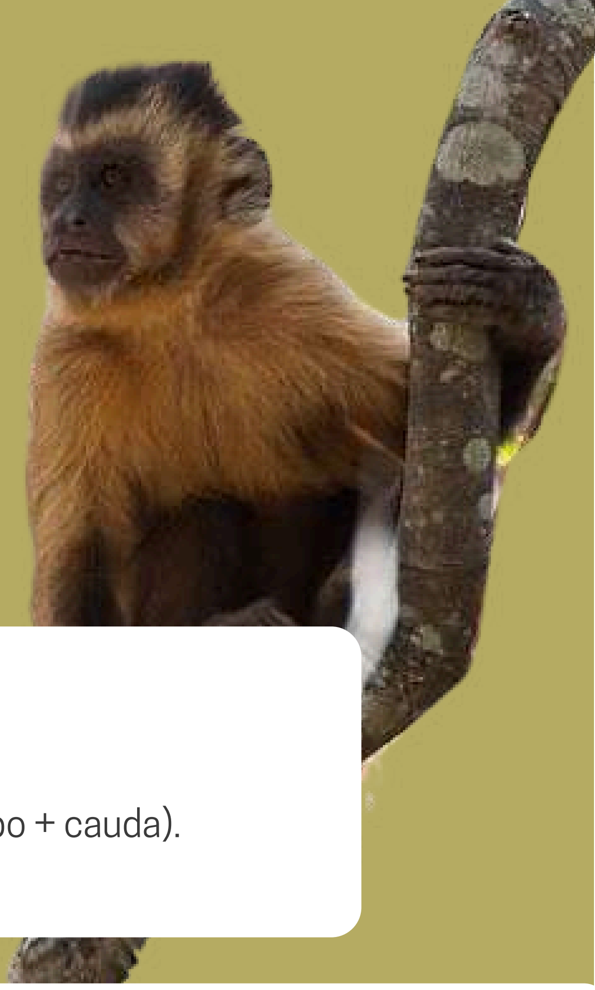

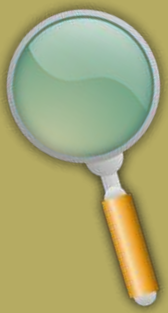

COMO EU SOU

- Peso entre 2 e 4 quilos.
- Tenho de 72 a 104 cm de tamanho (corpo + cauda).
- Vivo em grupos de 6 a 30 indivíduos.

Não é difícil me reconhecer: sou grande, tenho pelo amarronzado e fama de roubar as coisas das pessoas, mas isso não é verdade!
Minhas expressões faciais são inconfundíveis! Assim como vocês, nós usamos uma variedade de expressões faciais para a comunicação dentro do nosso grupo social.

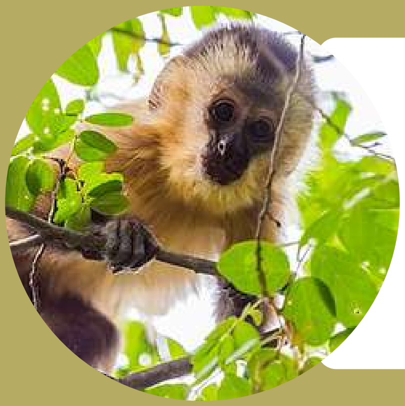

É DIFÍCIL ME ENCONTRAR

Sou muito adaptável e frequento muitos ambientes diferentes, desde florestas tropicais até alguns lugares bem secos e áridos. Em Belo Horizonte, eu frequento poucos locais, como a mata do Museu de História Natural e Jardim Botânico da UFMG.

ALIMENTAÇÃO

Sou onívoro, isso significa que gosto de comer uma grande variedade de alimentos: frutas, folhas, insetos, ovos e pequenos vertebrados.

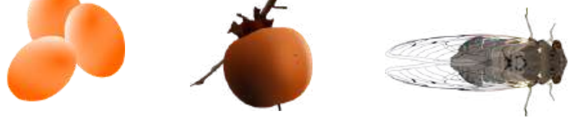

Assim como disse meu amigo mico-estrela, eu também tenho muita comida na mata. Então, não me alimente!

Quer saber mais sobre os primatas do Brasil?

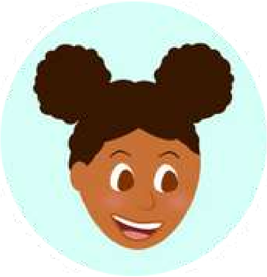

5 CURIOSIDADES SOBRE OS MICOS E MACACOS-PREGOS

1

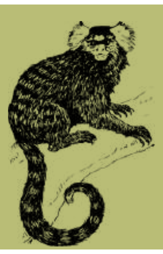

O mico-estrela é uma espécie invasora da Mata Atlântica

O Cerrado é o hábitat natural dos micos-estrela, portanto são considerados invasores em áreas de Mata Atlântica. Neste bioma, eles podem competir com espécies locais, o que cria desafios ecológicos.

2

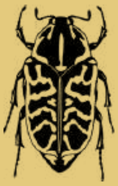

Macaco-prego e sua versatilidade

Os macacos-prego tem uma dieta muito diversificada, e uma grande capacidade de adaptação. Estas características os tornam um dos primatas mais bem-sucedidos em diferentes habitats.

3

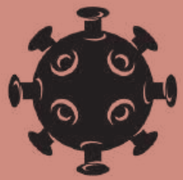

O herpes vírus humano é fatal para os primatas

O herpesvírus que causa doenças em humanos pode infectar os primatas e ser fatal. Alimentar primatas pode transmitir doenças, colocando suas vidas em risco. Por isso, não alimente e não toque nos primatas.

4

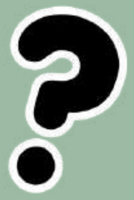

Você tem irmão gêmeo?

As fêmeas dos micos-estrela quando tem filhotinhos, sempre tem gêmeos, mas gêmeos não idênticos. Todos os indivíduos do grupo participam dos cuidados dos filhotes e é o pai quem carrega os filhotes nas costas!

5

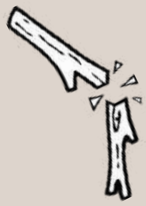

Macaco-prego e suas habilidades

Os macacos-prego são uma das poucas espécies de primatas que utilizam ferramentas, como galhos e pedras. Para se alimentar, eles quebram cocos e outras sementes com pedras, demonstrando uma habilidade cognitiva notável!

RISCOS AO INTERAGIR COM OS PRIMATAS

Vocês sabiam que quando vocês nos dão comida, podem acabar causando problemas para nossa saúde e até mesmo para a saúde de vocês?

Problemas para os primatas:

- Os alimentos que as pessoas comem não são adequados e saudáveis para os primatas, nem mesmo as frutas.
- Quando os primatas recebem comida nos parques, eles aprendem que esta é uma forma mais fácil de encontrar comida e não buscam pelo seu alimento natural.
- Quando os primatas comem alimentos que não fazem parte da sua dieta, eles podem ter doenças como obesidade, diabetes e deficiências nutricionais.

Problemas para os seres humanos:

- Quando os primatas aprendem a comer alimentos dos seres humanos, eles podem roubar comidas das nossas casas ou das pessoas que visitam os parques.
- Desta forma, eles podem machucar as pessoas, e eventualmente transmitir doenças. Também podem espalhar lixo e sujeira, o que pode atrair insetos e outros animais prejudiciais.

Por isso, é realmente importante que vocês não alimentem os primatas!

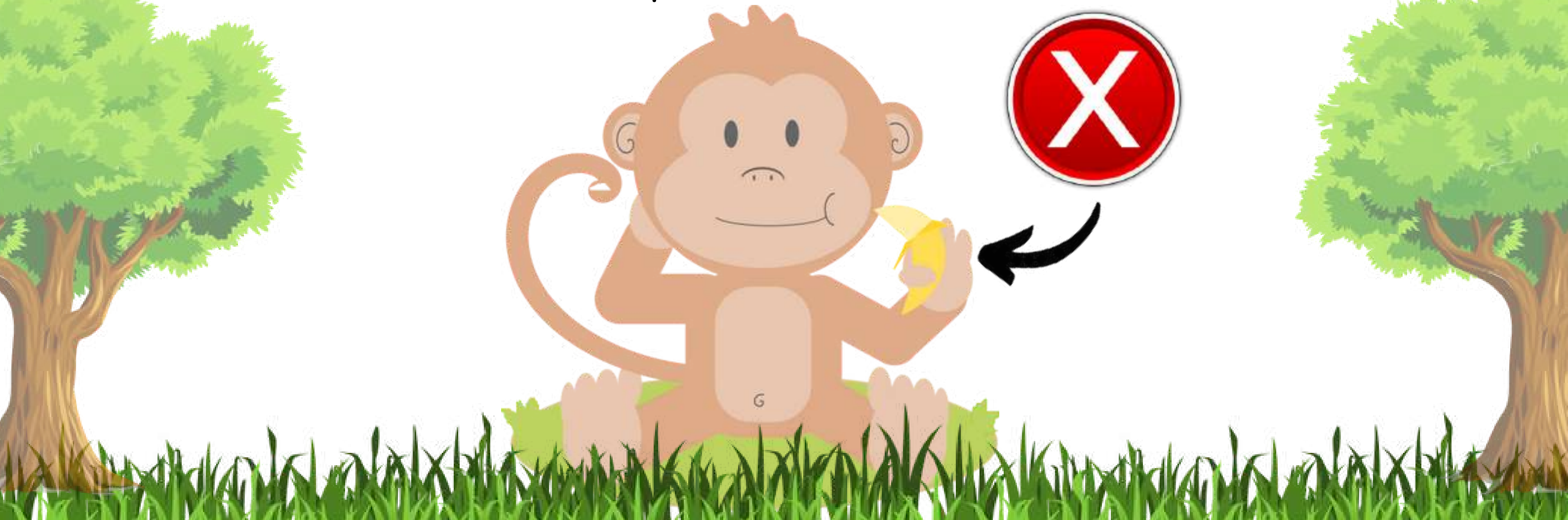

FEBRE
AMARELA

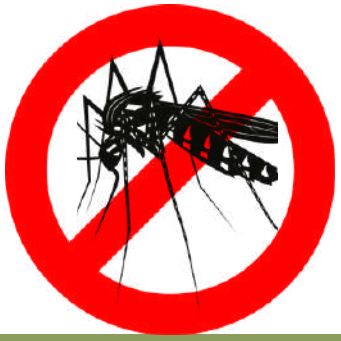

1. A febre amarela é uma doença grave, causada pelo vírus da febre amarela.

2. No Brasil, existe a febre amarela silvestre, quando o vírus é transmitido por mosquitos silvestres.

3. Mosquitos silvestres, chamados *Haemagogus* e *Sabethes*, podem transmitir o vírus da febre amarela para os primatas não humanos e para as pessoas.

4. Os primatas também são vítimas da febre amarela. Eles não transmitem o vírus para as pessoas.

5. ## Você sabia?

Quando um primata é encontrado morto ou doente por febre amarela, eles são nossos sentinelas, pois avisam que o vírus está por perto! Assim podemos nos proteger da doença.

Não maltrate os animais. Se você encontrar um primata morto ou doente, avise o Serviço de Controle de Zoonoses de sua cidade.

6. ## COMO POSSO ME PREVENIR?

A vacina é a principal ferramenta de prevenção e controle da febre amarela.

A vacina contra a febre amarela é gratuita pelo SUS.

Procure um posto de saúde e mantenha sua vacinação em dia.

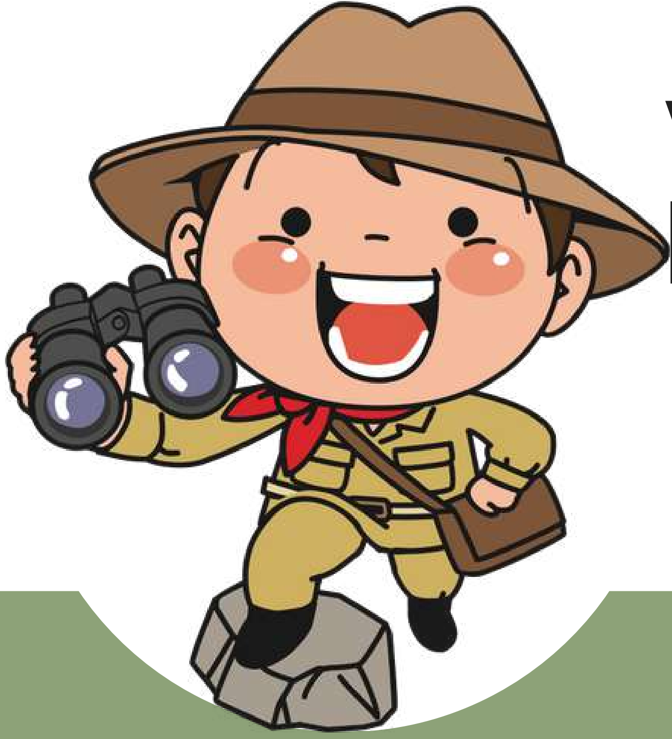

Viu um primata na NATUREZA?

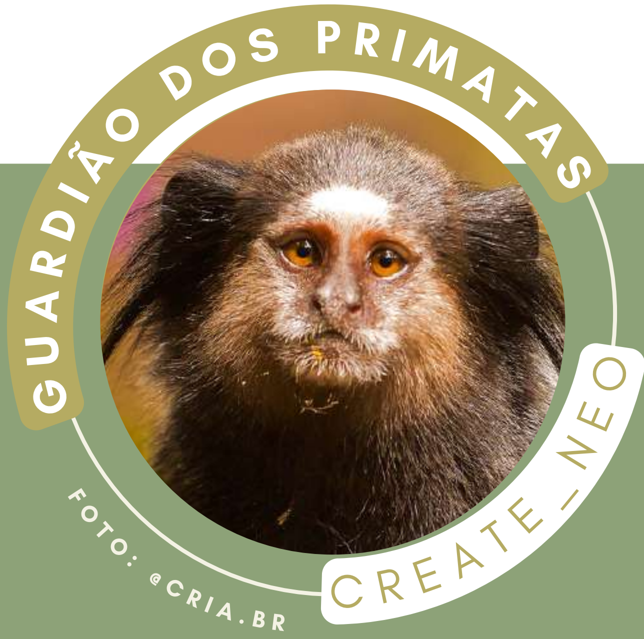

SEJA UM GUARDIÃO DOS PRIMATAS

- Mantenha distância dos animais.
- Não retire plantas ou coloque fogo nas matas.
- Não alimente os primatas, nem mesmo com frutas.
- Jogue o lixo na lixeira e não no chão.
- Tire somente fotos e sem flash.
- Se encontrar um animal ferido no parque, chame a gerência.

Achou um animal silvestre ferido ou morto? Não toque nele, ligue para:

Waita SOS animais silvestres
(31) 99462-4867

PM Ambiental
(31) 2123-1600/1635

Centro de controle de zoonoses
(31) 3277-7414

Bombeiros: 193

Guarda Municipal: 153

O mico está com fome

Os micos comem muitos alimentos na mata. Encontre o nome de três deles no nosso caça-palavras e ajude o mico a se alimentar!

A	G	O	M	A	C	I	C	G	H	E
T	B	F	M	L	V	R	N	Q	O	Q
O	L	Y	D	J	U	T	H	O	T	B
R	T	R	P	A	Z	G	R	R	O	V
G	R	I	L	O	S	M	E	I	R	A
S	L	I	B	A	R	A	T	A	A	B
F	E	L	Z	O	I	D	O	D	E	T

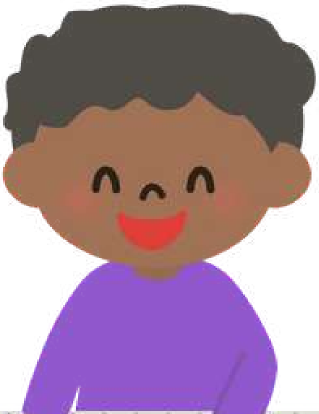

PALAVRAS CRUZADAS

Brinque e aprenda um pouco mais sobre os primatas brasileiros!

1. Meu nome científico é *Callithrix penicillata* e estou em muitos lugares em Belo Horizonte
2. O animal _______é aquele que se alimenta de uma variedade de alimentos, de origem vegetal ou animal.
3. Emissão de sons pelos primatas, usada para comunicação e, em espécies como o bugio, para marcar território.
4. Substância viscosa retirada de árvores, uma importante fonte alimentar para os micos.
5. Período do dia que os primatas são mais ativos.
6. Seiva branca e espessa extraída de árvores, consumida por micos, em algumas regiões.

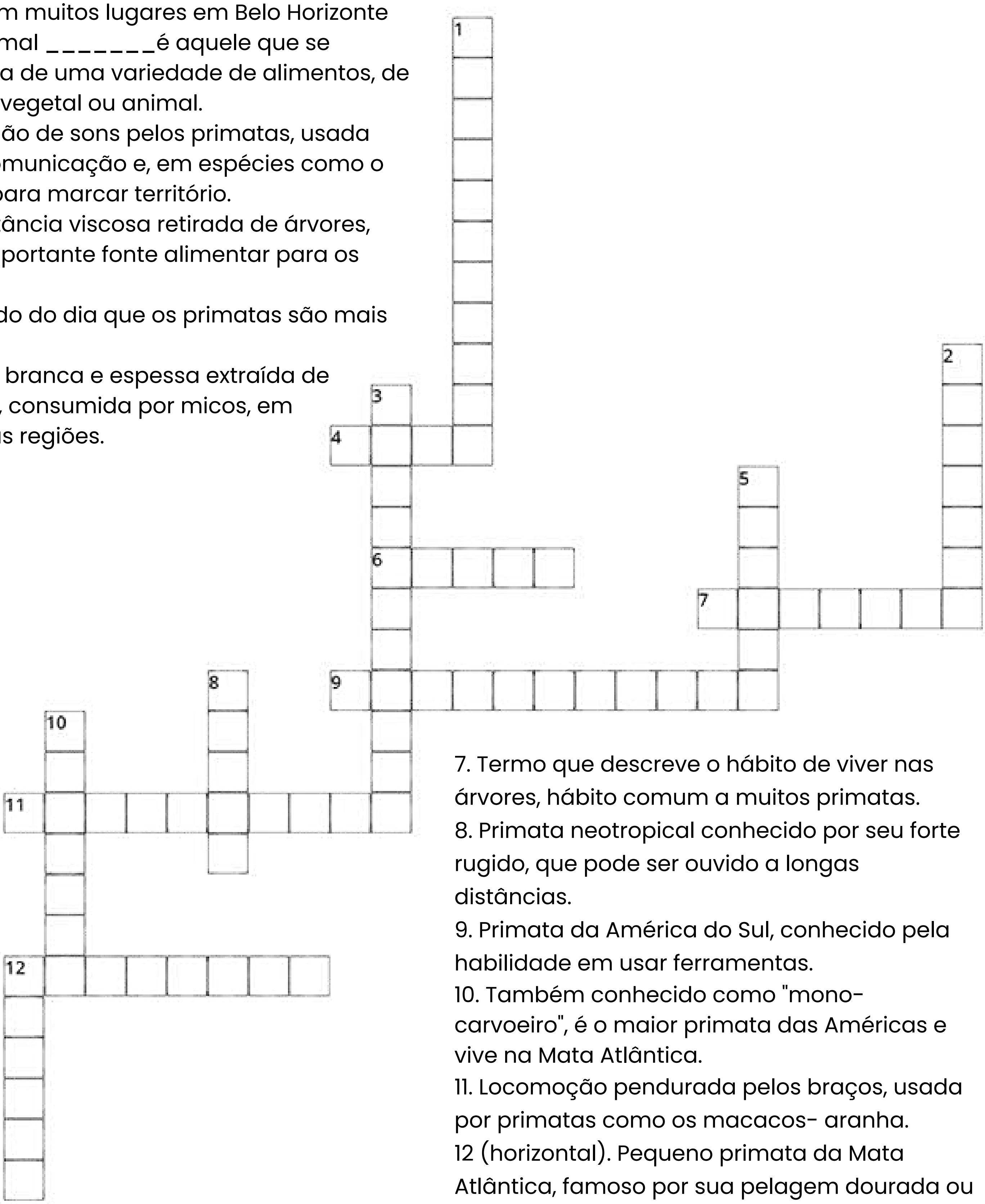

7. Termo que descreve o hábito de viver nas árvores, hábito comum a muitos primatas.
8. Primata neotropical conhecido por seu forte rugido, que pode ser ouvido a longas distâncias.
9. Primata da América do Sul, conhecido pela habilidade em usar ferramentas.
10. Também conhecido como "mono-carvoeiro", é o maior primata das Américas e vive na Mata Atlântica.
11. Locomoção pendurada pelos braços, usada por primatas como os macacos- aranha.

12 (horizontal). Pequeno primata da Mata Atlântica, famoso por sua pelagem dourada ou preta.

12 (vertical) . Nome genérico para vários primatas, muitos deles com cauda.

Respostas: 1. Mico-estrela, 2. onívoro, 3. vocalização, 4. goma, 5. diurno, 6. látex, 7. arbóreo, 8.bugio, 9.macaco-prego, 10. muriqui, 11. braquiação, 12 (horizontal). mico-leão, 12 (vertical). macaco.

Se interessou e quer saber mais sobre os primatas brasileiros?

Escaneie os QR Codes abaixo e tenha mais informações.

Sociedade Brasileira de Primatologia

Sociedade Brasileira de Mastozoologia

Livro A primatologia no Brasil

APOIO E REALIZAÇÃO

Mikaelly Frasson Biccas
Marcelle Alves de Oliveira
Daniel Jacob Circuncisão
Ana Maria de Oliveira Paschoal
Daniel Ambrósio Vilela
Betânia Paiva Drumond

IMAGENS USADAS:

Este guia foi elaborado utilizando uma combinação de fotografias (de Daniel Jacob e Eric Souz) e imagens disponíveis gratuitamente na Internet em termos de acesso livre ao abrigo da licença CC BY 4.0.

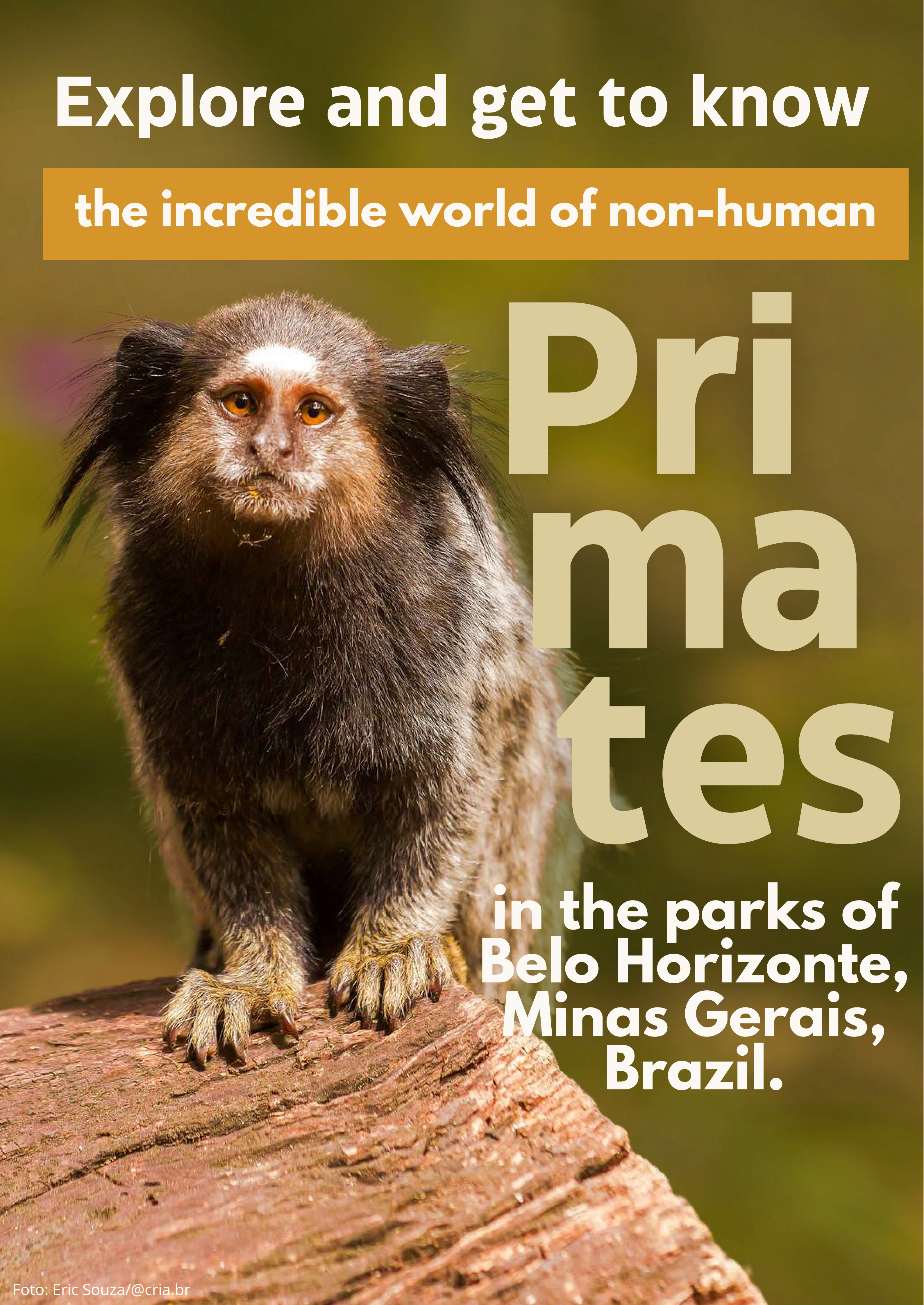
Explore and get to know
the incredible world of non-human
Primates
in the parks of
Belo Horizonte,
Minas Gerais,
Brazil.
Foto: Eric Souza/@cria.br

Preface

Welcome to the educational guide **"The Amazing World of Primates in urban parks of Belo Horizonte"**.

This guide was created to provide information about two fascinating non-human primate species found in Belo Horizonte: the star marmoset (Callithrix penicillata) and the capuchin monkey (Sapajus nigritus).

We will explore curiosities about the biology, ecology and behavior of these animals. We'll also look at the main threats and responsible practices for dwelling with these species in a respectful way. We hope you'll fall in love with primates as much as we have.

Mikaelly Frasson Biccas
Marcelle Alves de Oliveira
Daniel Jacob Circuncisão
Ana Maria de Oliveira Paschoal
Daniel Ambrósio Vilela
Betânia Paiva Drumond

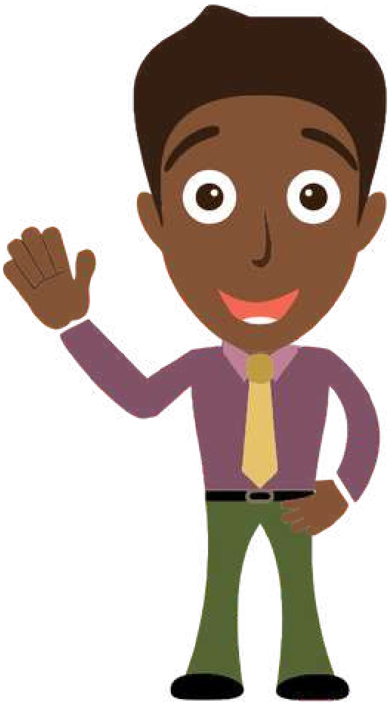

The urban parks

of Belo Horizonte city

The city of Belo Horizonte is located in Minas Gerais state in Southeast Brazil. Belo Horizonte stands out for its extensive green urban areas, covering more than 1,070 hectares and encompassing approximately 73 parks spread across its eight administrative regions. In addition to these parks, the city is home to various green spaces, including a botanical garden, a zoo, and agroecological experience centers.

Belo Horizonte hosts plant and animal species from the Cerrado and Atlantic Forest biomes. The biodiversity is remarkable: over 200 animal species inhabit these areas, alongside more than 1,000 plant species and numerous natural springs.

With the increasing urbanization, many of these parks are now in close proximity to residential and commercial areas. As a result, wildlife may come into contact with people—an encounter that can be both fascinating and potentially risky for both humans and animals.

In this brief guide, you are invited to explore the fascinating world of primates that inhabit the parks of Belo Horizonte and learn how to coexist with these species in a harmonious and respectful way.

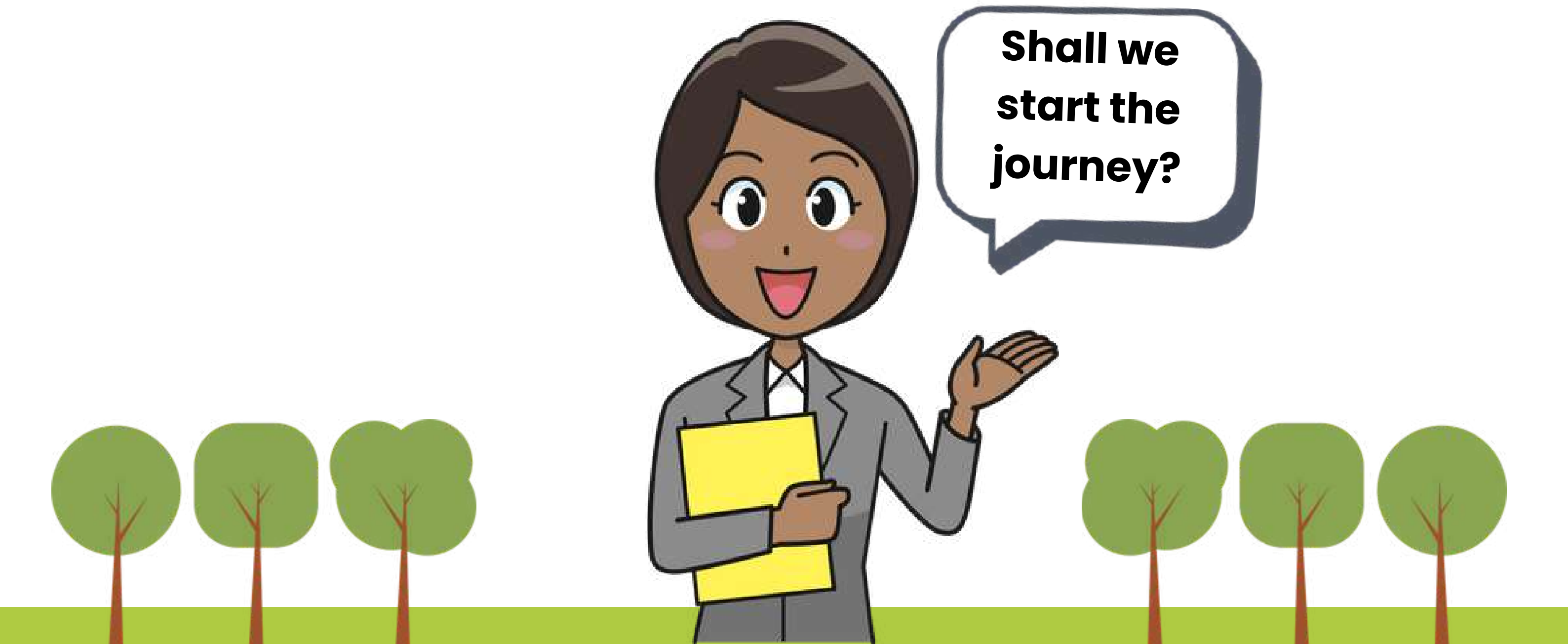

BLACK-TUFTED MARMOSET

Callithrix penicillata

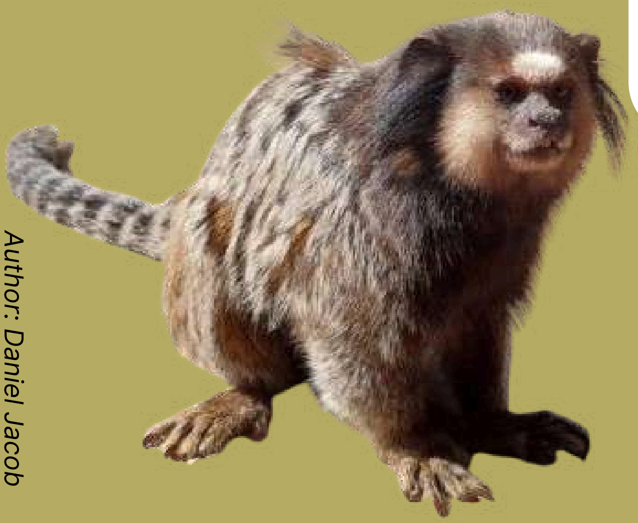

Author: Daniel Jacob

WHAT I LOOK LIKE

- I can grow up to 60 cm in length (body + tail).
- I weigh between 350 and 500 grams.
- I'm agile and sociable: I live in groups of 3 to 15 individuals.

WHY THIS NAME?

I'm the black-tufted marmoset, and I got this name because of the white, star-shaped patch on my forehead.

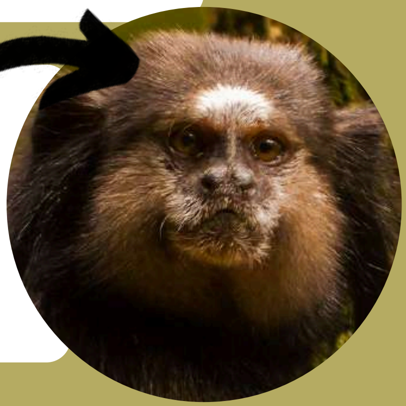

Author: Eric Souza

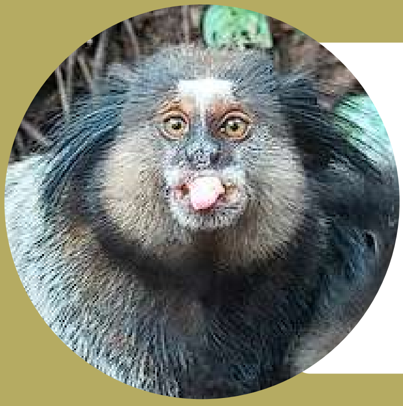

Author: Daniel Jacob

I'M EASY TO FIND

I'm highly adaptable—you've probably seen me in many places around the city! In my family, my mother always has twins, so I have lots of siblings!

DIET

I have a big appetite! I like to eat all kinds of insects, small vertebrates and invertebrates, wild fruits, and tree gum.

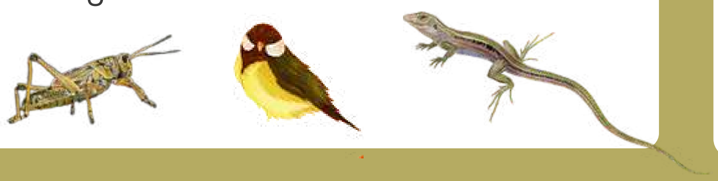

In nature, I never go hungry. That's why I don't need you to feed me! Your food isn't good for me, and soon, I'll explain why. But first, let me introduce you to my friends, the capuchin monkeys!

MONO CAPUCHINO

Sapajus sp.

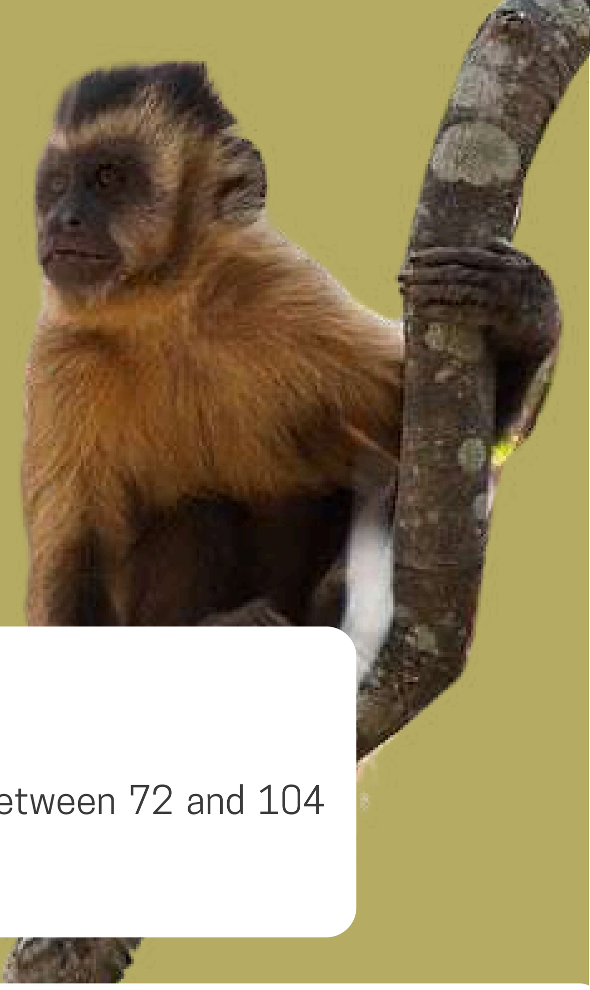

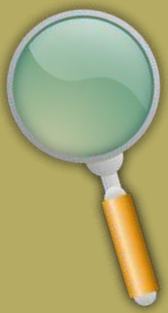

WHAT I LOOK LIKE

- I weigh between 2 and 4 kg.
- My body (including my tail) measures between 72 and 104 cm.
- I live in groups of 6 to 30 individuals.

I'm easy to recognize: I'm large, have brownish fur, and have a reputation for stealing things from people—but that's not true! My facial expressions are unmistakable! Just like you, we use a variety of facial expressions to communicate within our social group.

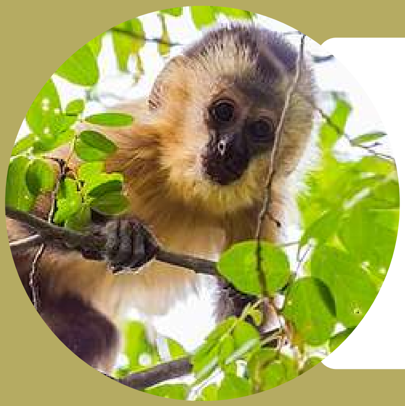

I'M HARD TO FIND

I'm very adaptable and live in many different environments, from tropical forests to dry, arid areas. In Belo Horizonte, I can only be found in a few places, such as the forested area of the UFMG Museum of Natural History and Botanical Garden.

DIET

I'm an omnivore, which means I eat a wide variety of foods as fruits, leaves, insects, eggs, and small vertebrates.

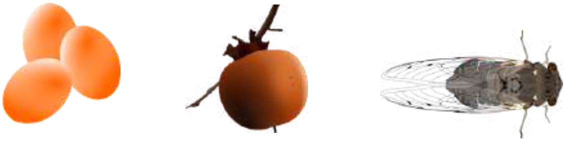

Just like my friend, the black-tufted marmoset, I have plenty of food in nature. So please, don't feed me.

Would you like to learn more about primates in Brazil?

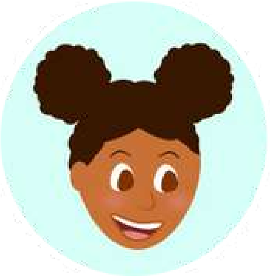

5 INTERESTING FACTS ABOUT MARMOSETS AND CAPUCHIN MONKEYS

1

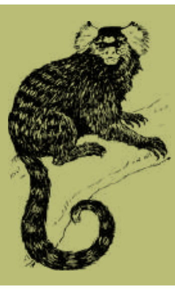

The Black-Tufted Marmoset Is an Invasive Species in the Atlantic Forest

The natural habitat of black-tufted marmosets is the Cerrado, the Brazilian Savanah. Therefore, they are considered invasive in the Atlantic Forest, where they can compete with native species, creating ecological challenges.

2

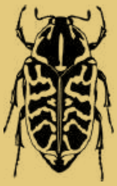

The Versatility of Capuchin Monkeys

Capuchin monkeys have a highly diverse diet and remarkable adaptability. These traits make them one of the most successful primates across different habitats.

3

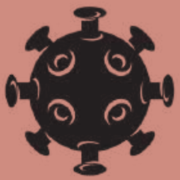

Human Herpesvirus Is Fatal to Primates

The herpesvirus that causes diseases in humans can infect primates and be fatal. Feeding primates can spread diseases, putting their lives at risk. That's why you only observe them from a distance and should never feed or touch them.

4

Do You Have a Twin?

Female black-tufted marmosets always give birth to twins — but not identical ones! In their groups, all members help care for the babies, and it's the father who carries them on his back!

5

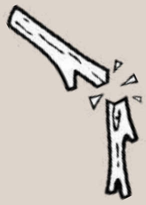

Capuchin Monkeys and Their Skills

Capuchin monkeys are among the few primate species that use tools, such as sticks and stones. To eat, they crack open coconuts and other seeds using rocks, showcasing remarkable cognitive abilities!

RISKS OF INTERACTING WITH PRIMATES

Did you know that feeding primates can harm their health—and even yours?

Risks for Primates:

- Human food, including fruit, is not suitable or healthy for primates.
- When primates receive food in parks, they learn to rely on it instead of searching for their natural and proper food.
- When primates eats inadequate food (such as food humans eat) can cause health issues such as obesity, diabetes, and nutritional deficiencies.

Risks for Humans:

- Once primates associate humans with food, they may steal food from houses or park visitors.
- This habit can lead to injuries, disease transmission, and even increased litter, attracting harmful insects and animals.

That's why it's so important not to feed the primates!

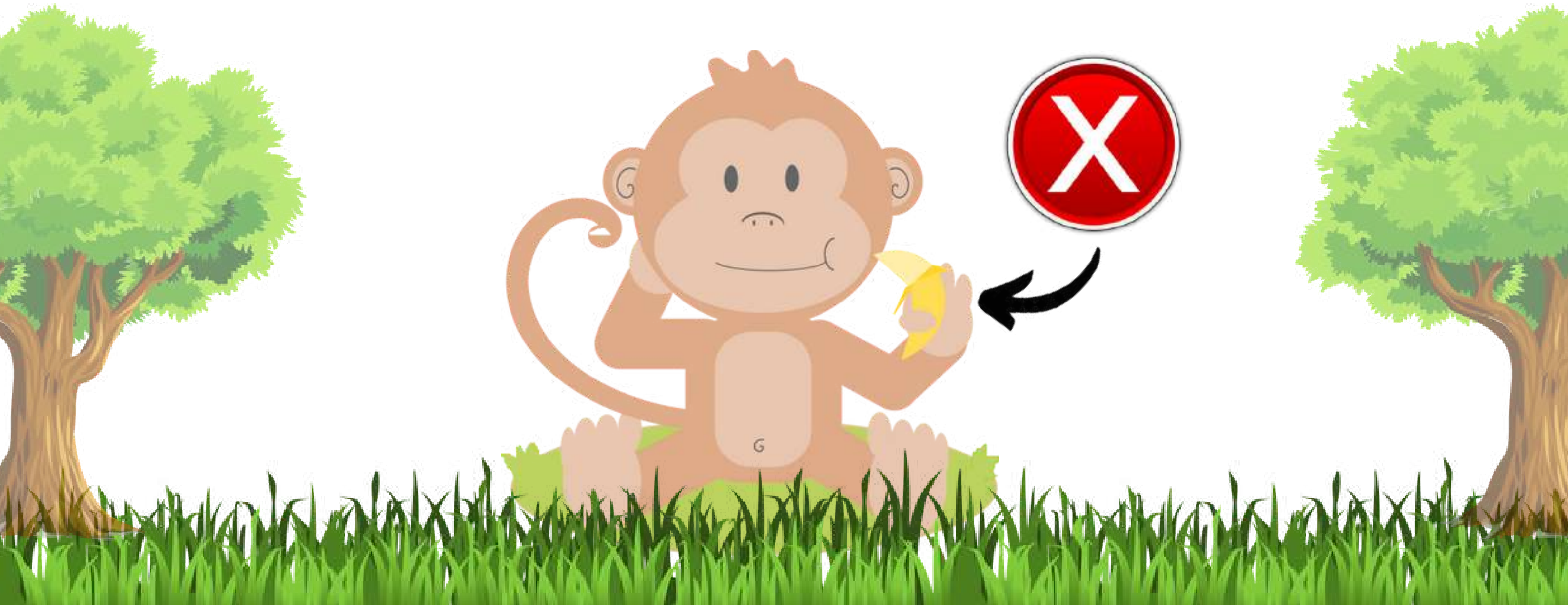

YELLOW

FEVER

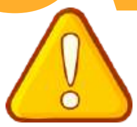

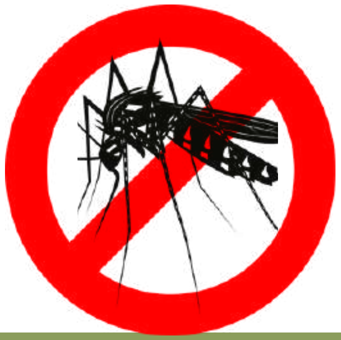

1. Yellow fever is a serious disease caused by the yellow fever virus.

2. In Brazil, sylvatic yellow fever is transmitted by forest-dwelling mosquitoes.

3. Mosquitoes of the Haemagogus and Sabethes genera can transmit the yellow fever virus to both primates and humans.

4. Primates are also victims of yellow fever. They do not transmit the virus to humans.

5. **Did you know?**

When a primate is found dead or sick due to yellow fever, they act as sentinels, warning us that the virus is present in that area! This information helps us take preventive measures against yellow fever.

Do not harm animals. If you find a dead or sick primate, contact the city’s Zoonosis Control Service.

6. **HOW CAN I PROTECT MYSELF FROM YELLOW FEVER?**

Vaccination is the main tool for preventing and controlling yellow fever.
The vaccine is free through the SUS (Brazil’s public health system).
If you are visiting Brazil, you should get vaccinated against yellow fever. Visit a health center and keep your vaccination up to date.

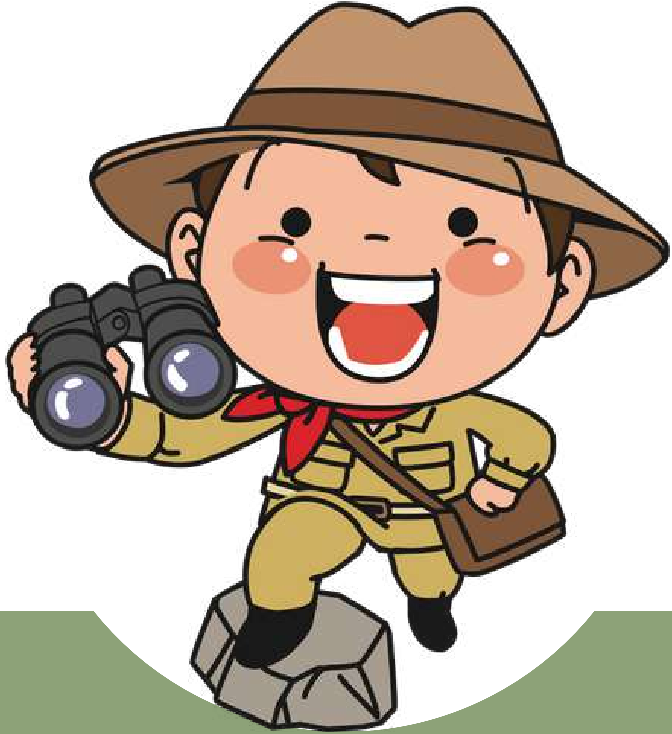

Did you spot a Primate

IN NATURE?

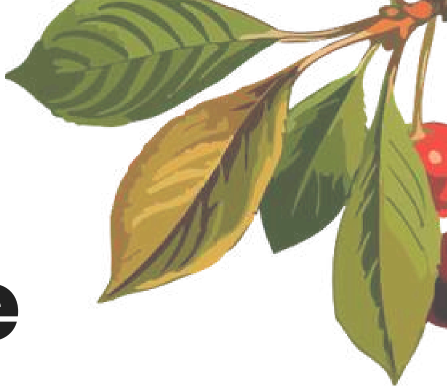

BE A PRIMATE GUARDIAN!

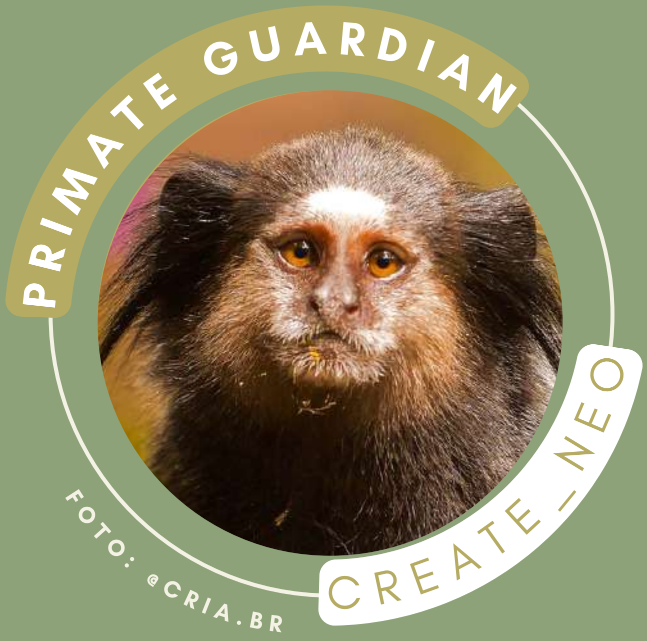

- Keep your distance from the animals.
- Do not remove plants or set fire in the forest.
- Do not feed the primates—not even with fruit.
- Dispose of trash properly—never litter.
- Take pictures but without flash.
- If you find an injured animal in the park, inform the park administration.

SUPPORT AND REALIZATION

Mikaelly Frasson Biccas
Marcelle Alves de Oliveira
Daniel Jacob Circuncisão
Ana Maria de Oliveira Paschoal
Daniel Ambrósio Vilela
Betânia Paiva Drumond

IMAGES USED:

This guide was created using a combination of photographs (by Daniel Jacob and Eric Souz) and freely available images from the Internet under open-access terms in accordance with the CC BY 4.0 license.

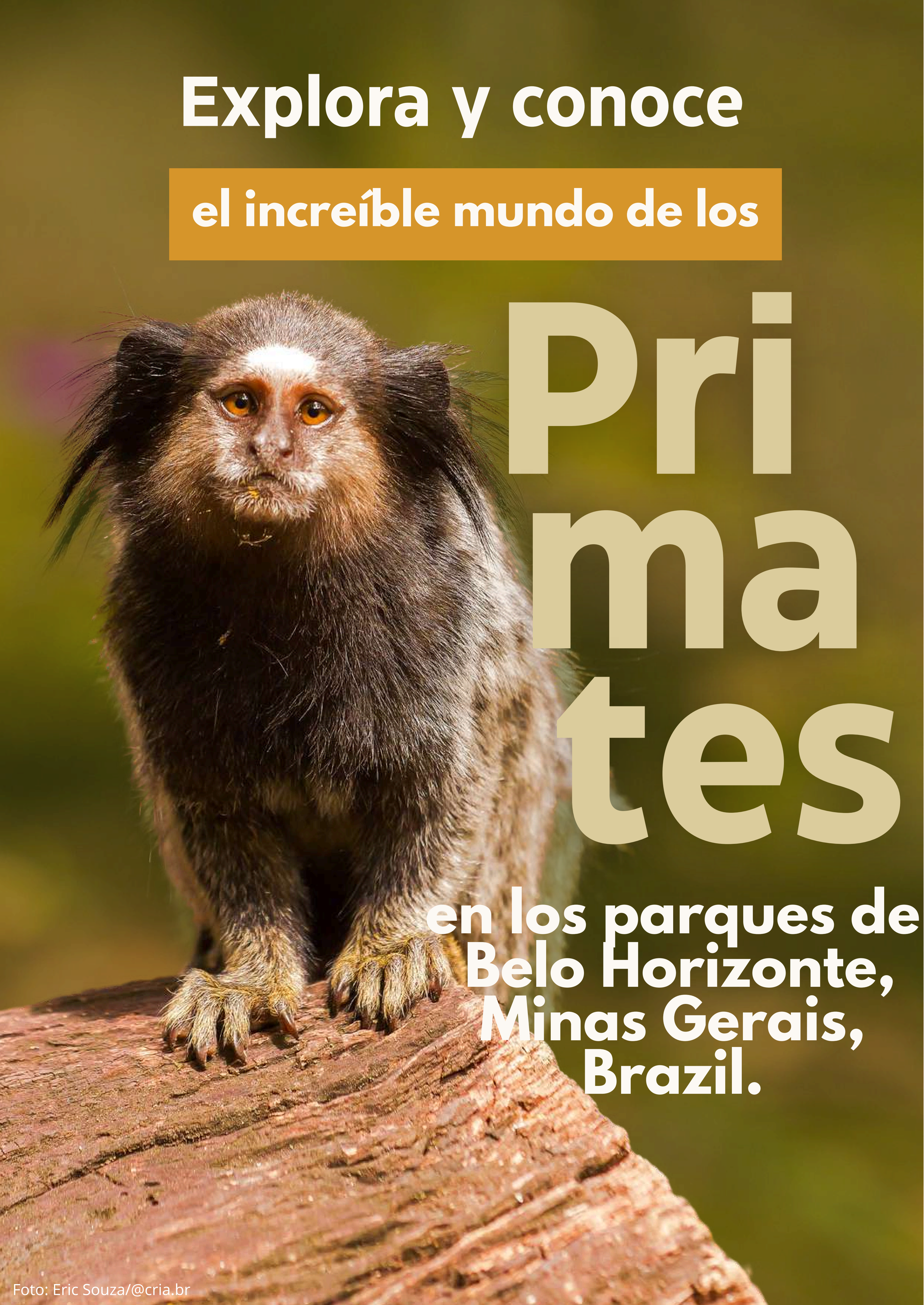

Foto: Eric Souza/@cria.br

Prefacio

Bienvenidos a la guía educativa **"El increíble mundo de los primates en los Parques Urbanos de Belo Horizonte".**

Esta guía fue creada para proporcionar información importante sobre dos fascinantes especies de primates que se encuentran en Belo Horizonte: el tití estrella (Callithrix penicillata) y el mono capuchino (Sapajus nigritus).

Exploraremos curiosidades sobre la biología, ecología y comportamiento de estos animales. También abordaremos las principales amenazas y las buenas prácticas para convivir con estas especies de manera respetuosa. ¡Esperamos que te enamores de los primates tanto como nosotros!

Mikaelly Frasson Biccas
Marcelle Alves de Oliveira
Daniel Jacob Circuncisão
Ana Maria de Oliveira Paschoal
Daniel Ambrósio Vilela
Betânia Paiva Drumond

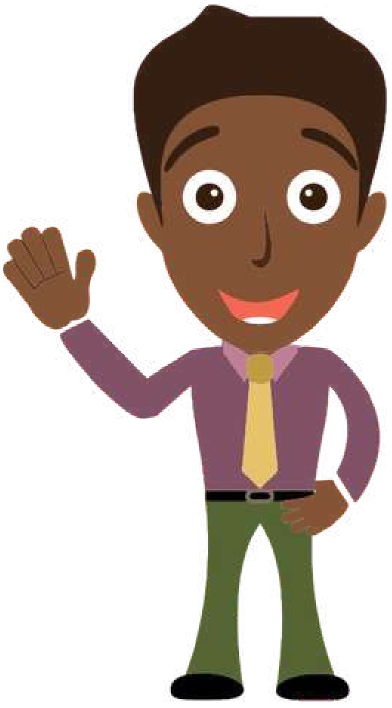

los parques urbanos

de Belo Horizonte

La ciudad de Belo Horizonte se destaca por contar con más de 1070 hectáreas de áreas verdes urbanas, que suman aproximadamente 73 parques distribuidos entre las 8 regiones de la ciudad. Además, la ciudad cuenta con diversas áreas verdes, como el jardín botánico, el zoológico y centros de vivencia agroecológica.

En Belo Horizonte encontramos especies de plantas y animales de los biomas Cerrado y Mata Atlántica. La diversidad es sorprendente: más de 200 especies de animales habitan estos espacios, junto con más de 1,000 especies vegetales y numerosas fuentes de agua.

Con el avance de la urbanización, muchos de estos parques se encuentran en proximidad directa con áreas residenciales y comerciales. Así, los animales silvestres pueden acercarse a las personas, lo que puede ser encantador, pero también representar riesgos tanto para las personas como para los animales.

En esta breve guía, te invitamos a explorar el fascinante universo de los primates que habitan los parques urbanos de Belo Horizonte y a descubrir cómo es posible convivir con estas especies de manera armoniosa y respetuosa.

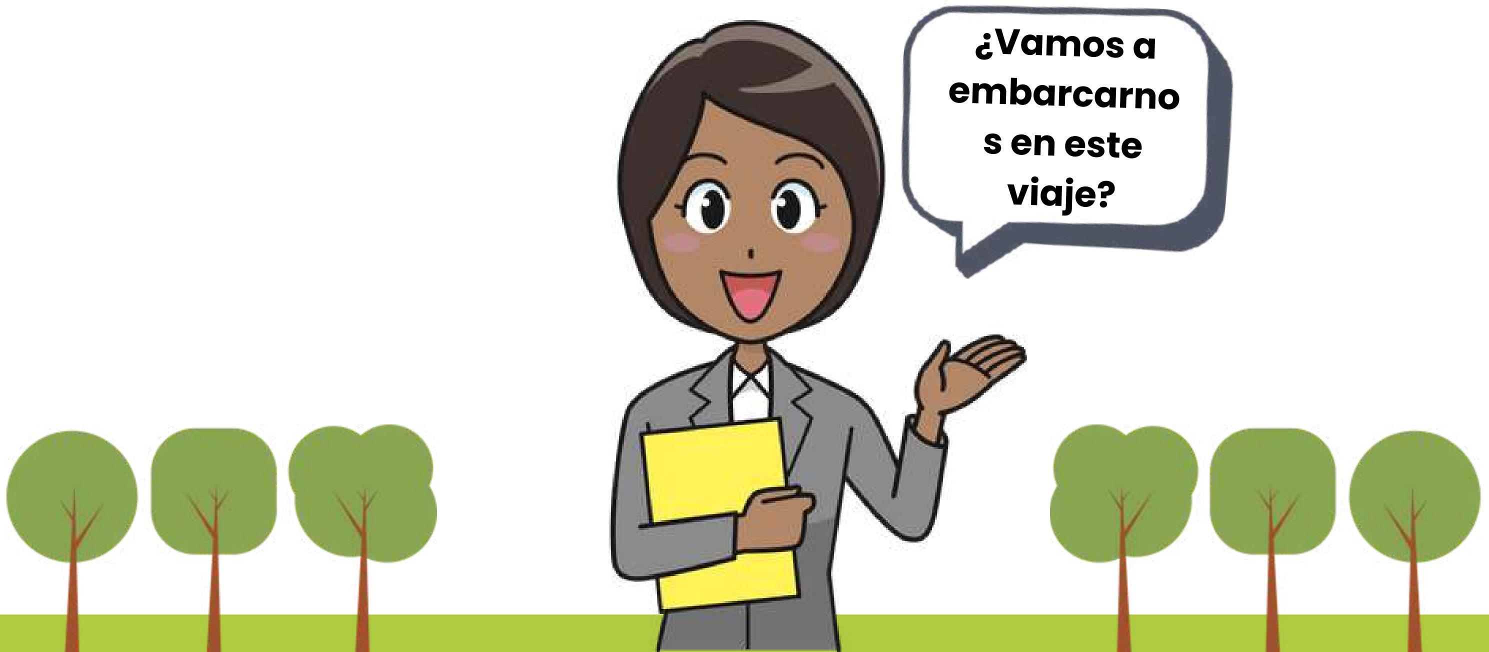

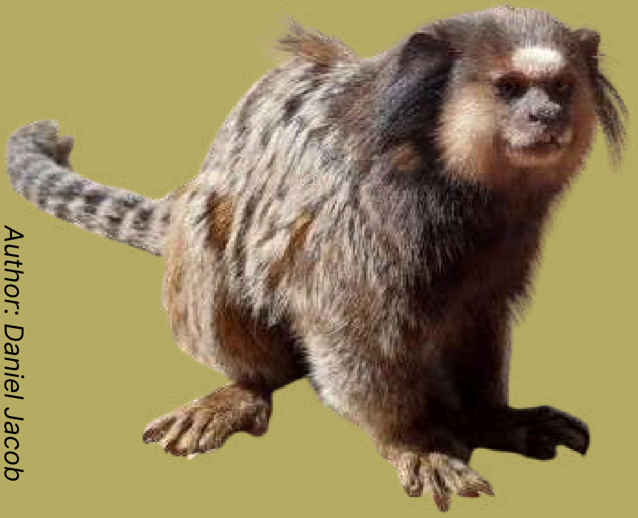

Author: Daniel Jacob

TITÍ ESTRELLA

Callithrix penicillata

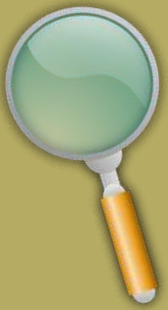

¿CÓMO SOY?

- Mido hasta 60 cm (cuerpo + cola).
- Peso entre 350 y 500 gramos.
- Soy ágil y sociable: vivo en grupos de 3 a 15 individuos.

¿POR QUÉ ESTE NOMBRE?

Soy el tití estrella. Recibo este nombre por la mancha blanca en forma de estrella en mi frente. ¡Sabías que también me llaman tití de penacho negro?

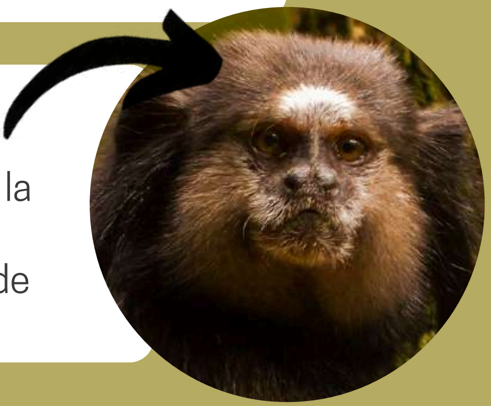

Author: Eric Souza

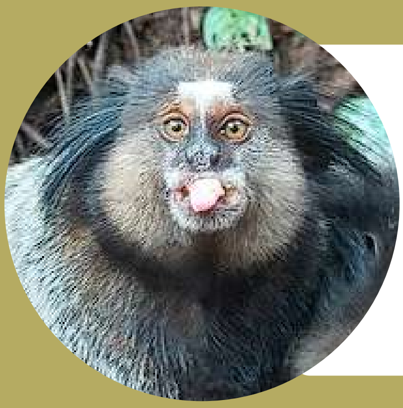

Author: Daniel Jacob

NO ES DIFÍCIL ENCONTRARME

Soy muy adaptable; probablemente ya me has visto en muchos lugares de nuestra ciudad. En mi familia, mi madre siempre tiene gemelos, ¡así que tengo muchos hermanos!

ALIMENTACIÓN

¡Tengo mucho apetito! Me gusta comer insectos, pequeños vertebrados e invertebrados, frutos silvestres y goma de los árboles.

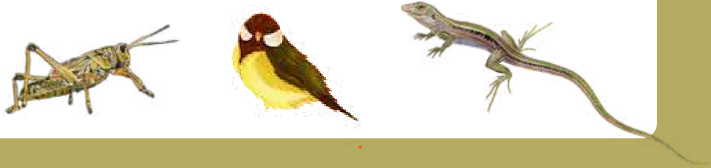

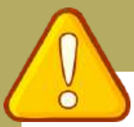

En la selva no paso hambre. Por eso, ¡no necesito que me ofrezcas tu comida! Tu comida no me hace bien, y pronto te contaré por qué. Pero antes, conocerás a mis amigos, los monos capuchinos.

MONO CAPUCHINO

Sapajus sp.

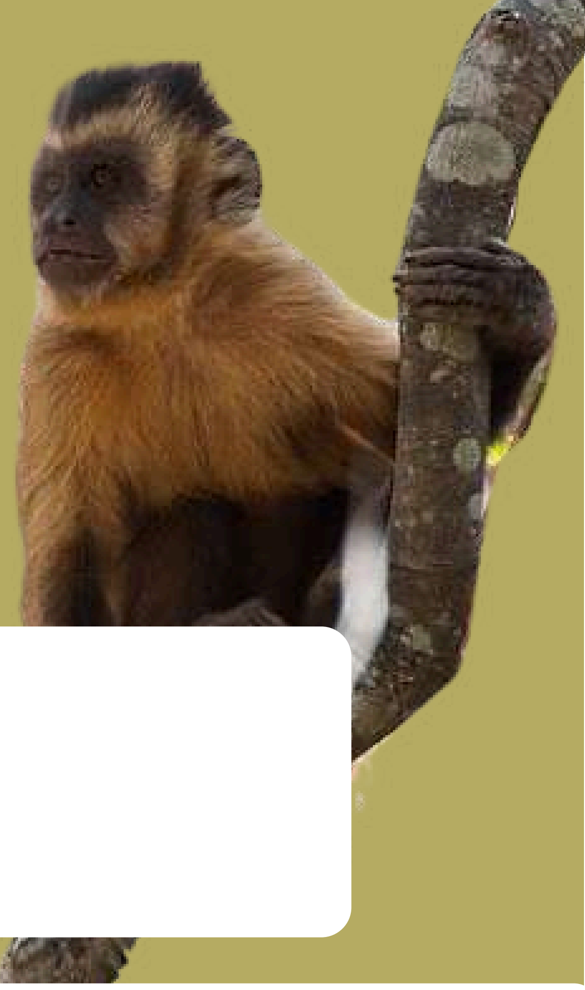

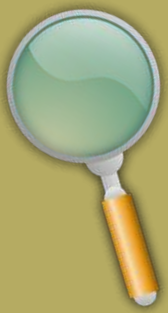

¿CÓMO SOY?

- Peso entre 2 y 4 kilos.
- Mido entre 72 y 104 cm (cuerpo + cola).
- Vivo en grupos de 6 a 30 individuos.

No es difícil reconocerme, soy grande, tengo pelaje marrón y fama de robar cosas a las personas, ¡pero eso no es verdad! Mis expresiones faciales son inconfundibles. Al igual que ustedes, utilizamos una variedad de expresiones faciales para comunicarnos dentro de nuestro grupo social.

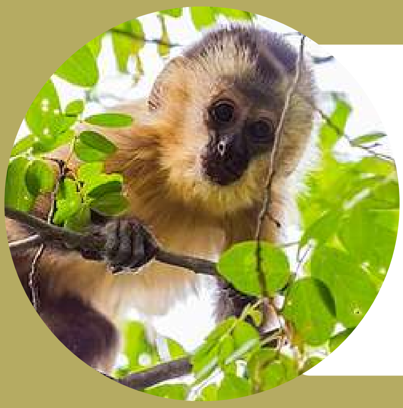

ES DIFÍCIL ENCONTRARME

Soy muy adaptable y frecuento muchos ambientes diferentes, desde selvas tropicales hasta lugares muy secos y áridos. En Belo Horizonte, habito pocos lugares, como el bosque del Museo de Historia Natural y Jardín Botánico de la UFMG.

ALIMENTACIÓN

Soy omívoro, lo que significa que me gusta comer una gran variedad de alimentos: frutas, hojas, insectos, huevos y pequeños vertebrados.

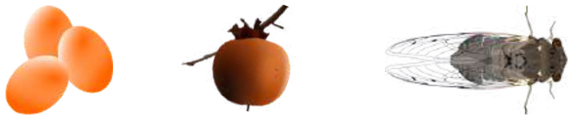

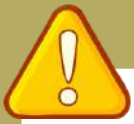

Al igual que dijo mi amigo tití estrella, yo también tengo mucha comida en la selva. ¡Así que no me alimentes!

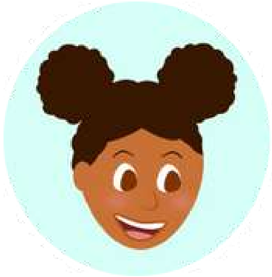

5 CURIOSIDADES SOBRE LOS TITÍES Y LOS MONOS CAPUCHINOS

1

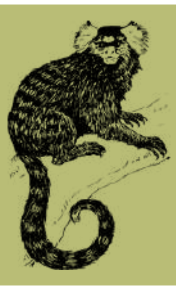

El tití estrella es una especie invasora en la Mata Atlántica.

2

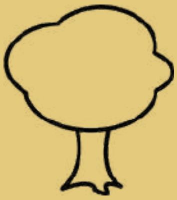

El mono capuchino es muy versátil y exitoso en diferentes hábitats.

3

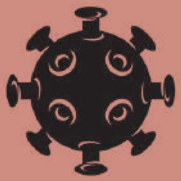

El herpesvirus humano es mortal para los primates.

4

Las hembras de los titíes estrella siempre tienen gemelos.

5

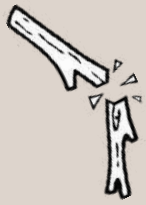

Los monos capuchinos utilizan herramientas para alimentarse.

RIESGOS AL INTERACTUAR CON LOS PRIMATES

¿Sabías que al darles comida puedes causar problemas para su salud y también para la tuya?

Problemas para los primates:

- La comida de los humanos no es saludable para ellos.
- Se vuelven dependientes de la comida fácil y dejan de buscar su alimento natural.
- Pueden desarrollar enfermedades como obesidad y diabetes.

Problemas para los humanos:

- Aprenden a robar comida y pueden lastimar a las personas.
- Pueden propagar basura y atraer plagas.

Por eso, ¡es muy importante no alimentar a los primates!

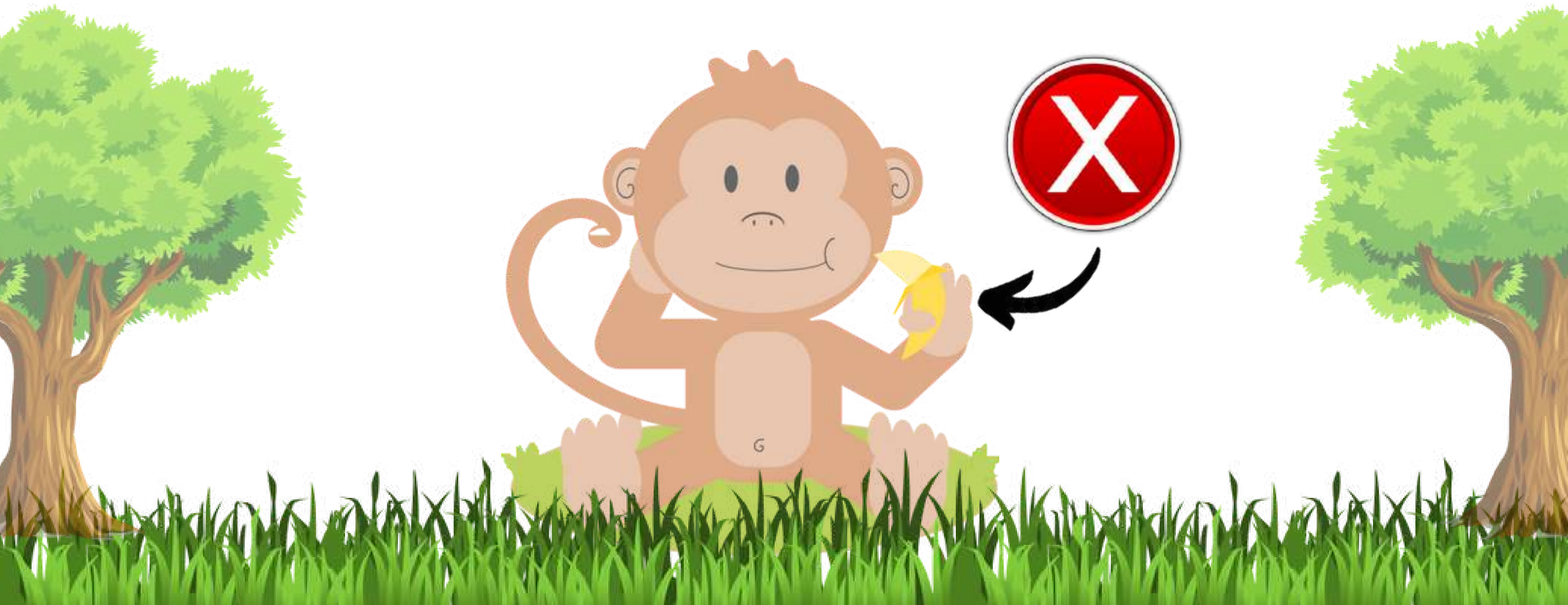

FIEBRE
AMARILLA

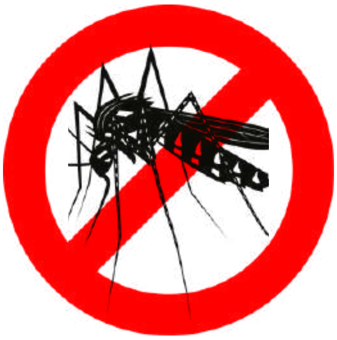

1. Es una enfermedad grave causada por un virus.

2. En Brasil, existe la fiebre amarilla silvestre transmitida por mosquitos.

3. Los mosquitos Haemagogus y Sabethes transmiten el virus.

4. Los primates también son víctimas, pero no transmiten el virus a los humanos.

5. Los primates son centinelas que nos alertan sobre la presencia del virus.

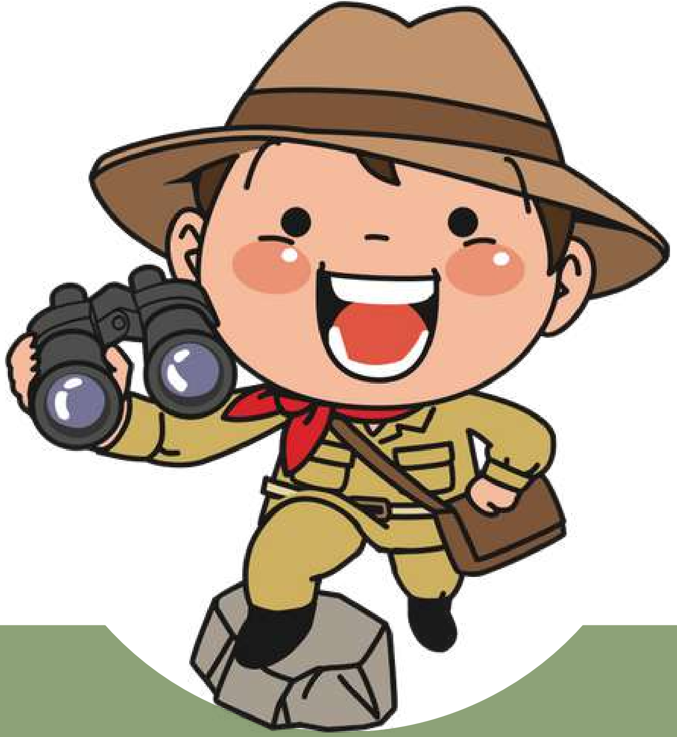

¿Viste un primate EN LA NATURALEZA?

¡SÉ UN GUARDIÁN DE LOS PRIMATES!

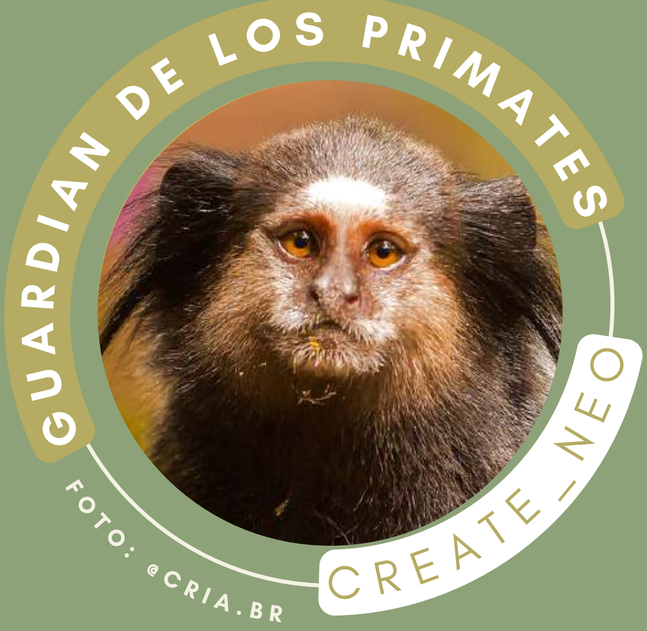

- Mantente a distancia.
- No arranques plantas ni provoques incendios.
- No los alimentes.
- No tires basura en el suelo.
- Solo toma fotos sin flash.
- Si ves un animal herido, avisa a la administración del parque.

APOYO Y REALIZACIÓN

Mikaelly Frasson Biccas
Marcelle Alves de Oliveira
Daniel Jacob Circuncisão
Ana Maria de Oliveira Paschoal
Daniel Ambrósio Vilela
Betânia Paiva Drumond

IMÁGENES UTILIZADAS:

Esta guía fue elaborada utilizando una combinación de fotografías (de Daniel Jacob y Eric Souz) e imágenes disponibles gratuitamente en Internet, bajo términos de acceso libre conforme a la licencia CC BY 4.0.
